# Bayesian nonlinear expectation for time series modelling and its application to Bitcoin

**DOI:** 10.1007/s00181-022-02255-z

**Published:** 2022-05-25

**Authors:** Tak Kuen Siu

**Affiliations:** Department of Actuarial Studies and Business Analytics, Macquarie Business School, Macquarie University, Sydney, NSW 2109 Australia

**Keywords:** Parametric time series modelling, Nonlinear expectations, Bayesian statistics, Girsanov’s transform, Drift and volatility uncertainties, Bitcoin, C22, C11, C58

## Abstract

This paper proposes a two-stage approach to parametric nonlinear time series modelling in discrete time with the objective of incorporating uncertainty or misspecification in the conditional mean and volatility. At the first stage, a reference or approximating time series model is specified and estimated. At the second stage, Bayesian nonlinear expectations are introduced to incorporate model uncertainty or misspecification in prediction via specifying a family of alternative models. The Bayesian nonlinear expectations for prediction are constructed from closed-form Bayesian credible intervals evaluated using conjugate priors and residuals of the estimated approximating model. Using real Bitcoin data including some periods of Covid 19, applications of the proposed method to forecasting and risk evaluation of Bitcoin are discussed via three major parametric nonlinear time series models, namely the self-exciting threshold autoregressive model, the generalized autoregressive conditional heteroscedasticity model and the stochastic volatility model.

**Supplementary Information:**

The online version contains supplementary material available at 10.1007/s00181-022-02255-z.

## Introduction

Human activities in taking time series observations, such as sunspot numbers, have a long history. An early inquiry for “formally” modelling time series may be tracked back to the seminal works of Yule (1927) and Slutsky (1927), where linear time series models were introduced to describe cyclical behaviour of time series observations. See, for example, Tong (1990, Page 18), and Hansen (2013, Page 400). Owing to the realization of limitations of linear time series models in explaining nonlinear real-world phenomena in the late 1970s, some major parametric nonlinear time series models were introduced in the late 1970s and the 1980s, such as the threshold autoregressive (TAR) model and its sub-class, namely the self-exciting TAR (SETAR) model, pioneered by Tong (1977, 1978, 1983), the autoregressive conditional heteroscedasticity (ARCH) model pioneered by Engle (1982), the stochastic volatility (SV) model of Taylor (1982, 1986), the generalized ARCH (GARCH) model in Bollerslev (1986) and Taylor (1986) and the Markov-switching autoregressive model in Hamilton (1989) (see also Tong (1983)). See Tong (2002) for further discussions.

Though many significant problems in time series modelling have been studied, it seems that model uncertainty may not have received as much attention as those “mainstream” topics in the subject. It is, however, that in certain modern practical applications of time series modelling, such as artificial intelligence and Bitcoin forecasting and risk analysis, model uncertainty may play a significant role. In economics and econometrics, where time series analysis has been widely applied, the significance of incorporating model uncertainty and the “worst-case” scenario approach to model uncertainty were discussed in the Prize Lecture of Nobel Prize in Economic Sciences by Hansen (2013). The key idea of the “worst-case” scenario approach is to introduce a family of alternative models from a reference or approximating model and make decisions in the “worst-case” scenario over the set of alternative models (see Hansen and Sargent (2007)). Studies on model uncertainty, from the economics perspective, may be traced back to the distinction between risk and uncertainty in the classic monograph by Knight (1921), (see, for example, Hansen (2013), Section 6), where risk and uncertainty were referred to the situations where the probability models are known and where they are unknown, respectively.

Nonlinear expectations provide a plausible way to describe model uncertainty. The notion of nonlinear expectations and their stochastic calculus were introduced in the seminal works by Peng (1997, 2004, 2006, 2019), where the *g*-expectations for incorporating drift uncertainty and the *G*-expectations for describing volatility uncertainty were introduced. The mathematics for the *G*-expectations are more complicated than the *g*-expectations in a continuous-time modelling framework since the former involves singular measure changes, while the latter only involves absolutely continuous measure changes. Discrete-time nonlinear expectations were introduced in Elliott (2017) and Elliott and Siu (2017) with the objective of providing a convenient way to incorporating both drift and volatility uncertainties in normally distributed random shocks using a discrete-time Girsanov’s transformation in, for example, Elliott et al. (1995) and Elliott and Madan (1998). Specifically, both drift and volatility uncertainties are incorporated by discrete-time nonlinear expectations with absolutely continuous measure changes. A key issue for implementing nonlinear expectations is how a set of probability models defining the nonlinear expectations might be chosen. Specifically, from the practical perspective, it seems desirable to specify the set of probability models so that certain probability levels describing credibility of the set of probability models may be assigned. However, this issue has yet not been well-addressed in Elliott (2017), Elliott and Siu (2017), and possibly, in some other literature on nonlinear expectations.

The purpose of this paper is to propose a two-stage approach to parametric nonlinear time series modelling in discrete time with the objective of incorporating model uncertainty or misspecification in the conditional mean and volatility, which are two key components in nonlinear time series modelling. At the first stage, a reference or approximating time series model is specified. Then the reference model is estimated from given data. At the second stage, Bayesian nonlinear expectations are introduced to incorporate model uncertainty or misspecification in prediction via specifying a family of alternative models. Specifically, closed-form Bayesian credible intervals characterizing model uncertainty or misspecification in the conditional mean and volatility are constructed using conjugate priors and residuals of the estimated approximating model from the first stage. It may be noted that the residuals contain information that is left over, which may be attributed to model misspecification in the conditional mean and volatility of the reference or approximating model estimated from the first stage. The misspecifications in the conditional mean and volatility are estimated using the closed-form Bayesian credible intervals, and discrete-time Girsanov’s transforms are used to introduce the family of alternative models from the reference or approximating model. Those alternative models have misspecifications in the conditional mean and volatility lying in the ranges specified by the respective Bayesian credible intervals. Using the closed-form Bayesian credible intervals, the Bayesian nonlinear expectations incorporating those alternative models in prediction are then constructed. The closed-form Bayesian credible intervals have the advantage that they provide a convenient way to construct the Bayesian nonlinear expectations so that probability levels are assigned to the nonlinear expectations. It may also be noted that the closed-form Bayesian credible intervals provide a feasible way to incorporate both a model user’s subjective view (or expert opinion) and the objective data in estimating misspecifications in the conditional mean and volatility. Indeed, under the Bayesian statistical framework, the “unknown” misspecifications in the conditional mean and volatility are considered random variables, and this may provide a natural way to characterize model uncertainties about the conditional mean and volatility. These uncertainties are called drift and volatility uncertainties. Using real data in a Bitcoin exchange rates series including some volatile periods which may be due to Covid 19, applications of the proposed approach to forecasting and risk evaluation of Bitcoin are discussed. Three major nonlinear time series models, namely the SETAR model, the GARCH model and the SV model, are used for illustrating the applications. Two major tail-based risk metrics, say value at risk (VaR) and expected shortfall (ES), are used for risk evaluation of Bitcoin. Through the lens of Bayesian nonlinear expectations, the impacts of model uncertainty or misspecification in the conditional mean and volatility on forecasting and risk evaluation of Bitcoin are examined. It is hoped that the approach may throw light on exploring the interplay among time series modelling, Bayesian theory, nonlinear expectations, model uncertainty and their applications.

The proposed approach may be related to the Bayesian approach to nonlinear time series and Bayesian econometrics. See, for example, Pole and Smith (1985) for Bayesian analysis of SETAR models and Koop (2003) for an excellent account on Bayesian econometrics. However, the key difference between them is that the proposed approach is a two-stage approach, where the reference model is specified and estimated in the first stage, and the second stage is concerned with prediction in the presence of model uncertainty or misspecification using the Bayesian nonlinear expectations over a set of alternative models. The worst-case scenario approach to model uncertainty in economic decision-making was introduced in discrete-time economic models in, for example, Cagetti et al. (2002), Hansen et al. (2002) and Hansen and Sargent (2007). However, their focuses, modelling structures and methods are different from those of the current paper. Siu and Yang (1999) and Siu et al. (2001) adopted a Bayesian approach to generating probability scenarios for evaluating coherent risk measures whose representations are related to nonlinear expectations, (see Artzner et al. 1999 and Rosazza Gianin 2006). However, Siu and Yang (1999) and Siu et al. (2001) determined a set of probability models defining the coherent risk measures using a set of prior distributions. Siu (2001) used Bayesian credible intervals for evaluating interval estimates for coherent risk measures for derivatives under a discrete-time binomial tree model. Frazier et al. (2020) considered model misspecification in approximate Bayesian computation. However, the focuses and modelling structures of Siu (2001) and Frazier et al. (2020) are different from those of the current paper.

The rest of the paper is structured as follows. A brief introduction to nonlinear expectations is provided in the next section. Section 3 presents parametric nonlinear time series models with drift and volatility uncertainties using discrete-time Girsanov’s transforms, Gaussian uncertain noises and nonlinear expectations. The Bayesian nonlinear expectations are constructed using Bayesian credible intervals in Sect. 4. The estimation, forecasting and risk evaluation procedures are discussed in Sect. 5. The applications of the proposed approach to Bitcoin forecasting and risk evaluation using real data are provided in Sect. 6. The impacts of drift and volatility uncertainties on Bitcoin forecasting and risk evaluation are also examined. Section 7 gives some concluding remarks. Some tables and figures are presented in Appendices I–II, which are included after the bibliography. Online Appendices A–G provide some technical details, derivations, constructions, discussions on potential links to other important Bayesian techniques and potential generalizations of the proposed two-stage approach.

## *g*-Expectations, *G*-expectations and model uncertainty

In this section, a brief review on two types of nonlinear expectations, namely the *g*-expectation and the *G*-expectation, and their links with model uncertainty or ambiguity is provided. Some technical details on these are presented in Online Appendix A. The *g*-expectation was introduced by Peng (1997), where the *g*-expectation was defined by the solution of a backward stochastic differential equation. The *g*-expectation describes model uncertainty or ambiguity about the drift of a process, which is also called the drift uncertainty. The *G*-expectation was also introduced by Peng (2006) and describes model uncertainty or ambiguity about the volatility of a process, which is called the volatility uncertainty.

The notion of nonlinear expectations is closely linked with the concept of coherent risk measures in Artzner et al. (1999). Indeed, as noted by Peng (2004, 2006), the notion of nonlinear expectations are equivalent to the concept of coherent risk measures. Rosazza Gianin (2006) established the link between conditional *g*-expectations and dynamic risk measures. The concepts of coherent risk measures and nonlinear expectations also play a fundamental role in the two-price conic finance in, for example, Cherny and Madan (2009) and Madan and Cherny (2010), where market cones generating two-price systems were defined by sets of acceptable risks. A (conditional) nonlinear expectation satisfying the sub-additivity property is called a (conditional) sublinear expectation. A (conditional) expectation satisfying the super-additivity property is called a (conditional) superlinear expectation.

In a continuous-time modelling framework, a conditional *g*-expectation is defined by the solution of a backward stochastic differential equation. See, for example, Chen and Epstein (2002), where the conditional *g*-expectation was used to discuss a stochastic differential utility with multiple priors in the presence of model uncertainty or ambiguity in continuous time. The specification of a family of alternative models with the drift uncertainty described by the conditional *g*-expectation involves absolutely continuous probability measures. Specifically, the family of alternative models can be specified by applying Girsanov’s transforms for changing probability measures in continuous time.

The *G*-expectation was motivated by some works on option valuation with volatility uncertainty. Boyle and Ananthanarayanan (1977) introduced the use of Bayesian statistics to estimate volatility for option valuation, where Bayesian conjugate prior was used to capture the estimation risk of volatility and to construct interval estimates for option prices. Avellaneda et al. (1995) and Lyons (1995) introduced volatility uncertainty to option valuation and hedging, where the “uncertain” volatility was supposed to lie in a bounded interval and a convex region, respectively. Fouque and Ren (2014) considered the use of a conditional *G*-expectation in continuous time to study option valuation under volatility uncertainty. In fact, in a continuous-time modelling framework, the specification of alternative models with the volatility uncertainty described by the conditional *G*-expectation involves singular probability measures. Consequently, it is more complicated than the specification of a family of alternative models with the drift uncertainty described by the conditional *g*-expectation. However, in a discrete-time modelling framework, both the drift and volatility uncertainties can be specified by a discrete-time Girsanov’s transform as in Elliott (2017) and Elliott and Siu (2017). Indeed, in the discrete-time situation, only probability measures that are absolutely continuous to the reference probability measure are used to define a family of alternative models in the presence of both the drift and volatility uncertainties.

## Parametric nonlinear time series models with drift and volatility uncertainties

Two general parametric nonlinear time series models incorporating uncertainties in the conditional mean (or drift) and volatility are introduced using discrete-time Girsanov’s transforms, Gaussian uncertain noises and nonlinear expectations. The first model is a nonlinear autoregressive model with conditional heteroscedasticity which includes the SETAR model and the GARCH model. The second model is a general specification which includes the first model and a product process. Since the product process includes the SV model, the second model also includes the SV model. The two models are introduced in accordance with the two-stage approach. At the first stage, reference or approximating models^1^ are specified. At the second stage, discrete-time Girsanov’s transforms are used to introduce Gaussian uncertain noises and sets of alternative models from the reference models. Then the (conditional) nonlinear expectations which incorporate the sets of alternative models in prediction are defined, and some motivations for introducing the Bayesian nonlinear expectations for prediction in the second stage are discussed. The idea of using (Gaussian) uncertain noises and nonlinear expectations in filtering and estimation of hidden Markov models appeared in Elliott (2017), where discrete-time Girsanov’s transforms in, for example, Elliott et al. (1995) and Elliott and Madan (1998), were used to introduce uncertain noises and the respective family of alternative models. The work of Elliott (2017) was then extended to the filtering and estimation of hidden Markov-modulated stochastic volatility models with drift and volatility uncertainties in Elliott and Siu (2017).

A complete probability space $(\Omega , \mathcal{F}, \mathbb {P})$ is considered, where $\mathbb {P}$ is a reference probability measure corresponding to an approximating model. A time index set $\mathbb {T}$ is defined as $\{ 1, 2, \ldots , T \}$, where *T* is the overall number of observations in time series data and future time steps in prediction. Let $\{ X_t \}_{ t \in \mathbb {T}}$ be a real-valued time series process,^2^ or stochastic process, defined on $(\Omega , \mathcal{F}, \mathbb {P})$. In the sequel, the first and second models for $\{ X_t \}_{t \in \mathbb {T}}$ are described in Sects. 3.1 and 3.2, respectively.

### A nonlinear autoregressive model with heteroscedasticity

Let $\{ \epsilon _t \}_{t \in \mathbb {T}}$ be a sequence of independent and identically distributed (i.i.d.) standard normal random variables under the reference probability measure $\mathbb {P}$. That is, under $\mathbb {P}$, $\epsilon _t \sim N (0, 1)$, for each $t \in \mathbb {T}$. It is supposed that under $\mathbb {P}$, $\{ X_t \}_{t \in \mathbb {T}}$ follows the reference parametric nonlinear time series model:

3.1 $$\begin{aligned} X_t = f (X_{t-1}, X_{t-2}, \ldots , X_{t-p}) + g (X_{t-1}, X_{t-2}, \ldots , X_{t - q}) \epsilon _t, \end{aligned}$$

where $f : \Re ^p \rightarrow \Re$ and $g : \Re ^q \rightarrow \Re$ are two given measurable functions; *p* and *q* are two positive integers. It is assumed, for simplicity, that $g > 0$. The reference model in Eq. (3.1) is a nonlinear autoregressive model with conditional heteroscedasticity, which incorporates some important parametric nonlinear time series models such as the SETAR model and the GARCH model. It is assumed that the initial observations $\{ X_{- \max (p, q)}, X_{- \max (p, q) + 1}, \ldots , X_0 \}$ are given. When *f* is a linear function and *g* is a constant function, the model in Eq. (3.1) becomes a linear time series model with homoscedastic errors.

A discrete-time Girsanov’s transform is now employed to construct a family of probability measures equivalent to the reference measure $\mathbb {P}$. The family of equivalent probability measures generates Gaussian uncertain noises with drift and volatility uncertainties and the respective alternative models.

Let $\varvec{\theta }:= (\mu , \sigma )^{\prime } = (\mu (\varvec{\theta }), \sigma (\varvec{\theta }))^{\prime } \in \Re \times \Re _+$, where $\mu$ may be interpreted as misspecification in the conditional mean (or drift) of the reference model and $\sigma$ may be interpreted as misspecification in the volatility of the reference model. By definition, $\mu$ is considered the first coordinate $\mu (\varvec{\theta })$ of $\varvec{\theta }$, while $\sigma$ is regarded as the second coordinate $\sigma (\varvec{\theta })$ of $\varvec{\theta }$. If $\mu = 0$, there is no misspecification in the drift of the reference model. Likewise, if $\sigma = 1$, there is no misspecification in the volatility of the reference model. In practice, the misspecifications in the drift and volatility may not be known. Consequently, $\mu$ and $\sigma$ may be regarded as “uncertain” parameters. One may describe these “uncertain” parameters by specifying the ranges in which those parameters lie. Mathematically, this may be done by considering the following product intervals:

3.2 $$\begin{aligned} \varvec{\Theta }:= [ \mu ^-, \mu ^+ ] \times [\sigma ^-, \sigma ^+], \end{aligned}$$

for some $\mu ^-, \mu ^+ \in \Re$ and $\sigma ^-, \sigma ^+ \in \Re _+$ with $\mu ^- < \mu ^+$ and $\sigma ^- < \sigma ^+$. Then, the family $\{ (\mu (\varvec{\theta }), \sigma (\varvec{\theta })) | \varvec{\theta }\in \varvec{\Theta }\}$ may be used to describe the “uncertain” parameters $(\mu , \sigma )$ and to characterize drift and volatility uncertainties.

Let $\phi _{\mu , \sigma } (x)$ denote the probability density function (pdf) of a normal distribution $N (\mu , \sigma ^2)$ with mean $\mu$ and variance $\sigma ^2$. Write $\phi (x)$ for $\phi _{0, 1} (x)$. Let $\mathbb {F}^X$ be the $\mathbb {P}$-augmentation of the natural filtration $\{ \mathcal{F}^X_t \}_{t \in \mathbb {T}}$ generated by the time series process $\{ X_t \}_{t \in \mathbb {T}}$. That is, for each $t \in \mathbb {T}$, $\mathcal{F}^X_t$ is the $\mathbb {P}$-completed $\sigma$-field generated by the observations $\{ X_1, X_2, \ldots , X_t \}$. For each $\varvec{\theta }\in \varvec{\Theta }$, let $\{ \lambda _t (\varvec{\theta }) \}_{t \in \mathbb {T}}$ be an $\mathbb {F}^X$-adapted process on $(\Omega , \mathcal{F}, \mathbb {P})$ defined by:

3.3 $$\begin{aligned} \lambda _t (\varvec{\theta }) := \frac{\phi _{\mu , \sigma } (\epsilon _t)}{\phi (\epsilon _t)} = \frac{\phi (\frac{\epsilon _t - \mu }{\sigma } )}{ \sigma \phi (\epsilon _t)}. \end{aligned}$$

Consider, for each $\varvec{\theta }\in \varvec{\Theta }$, the following $\mathbb {F}^X$-adapted process $\{ \Lambda _t (\varvec{\theta }) \}_{t \in \mathbb {T}}$:

3.4 $$\begin{aligned} \Lambda _t (\varvec{\theta }) := \prod ^{t}_{k = 1} \lambda _k (\varvec{\theta }). \end{aligned}$$

It is known that for each $\varvec{\theta }\in \varvec{\Theta }$, $\{ \Lambda _t (\varvec{\theta }) \}_{t \in \mathbb {T}}$ is an $(\mathbb {F}^{X}, \mathbb {P})$-martingale, and so ${\text{ E }} [\Lambda _t (\varvec{\theta })] = 1$. Consequently, it can be used as a density process for changing probability measures on $\mathcal{F}^X_{T}$, and a new probability measure $\mathbb {P}^{\varvec{\theta }}$ equivalent to $\mathbb {P}$ on $\mathcal{F}^X_{T}$, for each $\varvec{\theta }\in \varvec{\Theta }$, can be defined as:

3.5 $$\begin{aligned} \frac{{\mathrm {d}} \mathbb {P}^{\varvec{\theta }}}{{\mathrm {d}} \mathbb {P}} \bigg |_{\mathcal{F}^X_{T}} := \Lambda _{T} (\varvec{\theta }). \end{aligned}$$

Using a discrete-time Girsanov’s theorem, (see, for example, Elliott et al. (1995) and Elliott and Siu (2017)), for each $\varvec{\theta }\in \varvec{\Theta }$, under a new probability measure $\mathbb {P}^{\varvec{\theta }}$, a family $\{ \epsilon _t (\varvec{\theta }) \}_{t \in \mathbb {T}}$ of random variables defined by putting:

3.6 $$\begin{aligned} \epsilon _t (\varvec{\theta }) := \frac{\epsilon _t - \mu }{\sigma }, \end{aligned}$$

is a sequence of i.i.d. standard normal random variables. That is, under $\mathbb {P}^{\varvec{\theta }}$,

$$\begin{aligned} \epsilon _1 (\varvec{\theta }) , \epsilon _2 (\varvec{\theta }) , \ldots , \epsilon _T (\varvec{\theta }) \overset{i.i.d.}{\sim } N(0, 1). \end{aligned}$$

Recall that the family $\{ (\mu (\varvec{\theta }), \sigma (\varvec{\theta })) | \varvec{\theta }\in \varvec{\Theta }\}$ may be used to describe the “uncertain” parameters $(\mu , \sigma )$ for drift and volatility misspecifications. For each $\varvec{\theta }\in \varvec{\Theta }$, let $\epsilon (\varvec{\theta }) := \{ \epsilon _t (\varvec{\theta }) \}_{t \in \mathbb {T}}$, which is a sequence of i.i.d. standard normal random variables under $\mathbb {P}^{\varvec{\theta }}$. Then the Gaussian uncertain noises are defined by the family $\{ \epsilon (\varvec{\theta }) | \varvec{\theta }\in \varvec{\Theta }\}$ of sequences of normal random variables indexed by $\varvec{\Theta }$ in Eq. (3.2), say the product intervals in which the “uncertain” parameters $(\mu , \sigma )$ lie. It may be noted that the Gaussian uncertain noises $\{ \epsilon (\varvec{\theta }) | \varvec{\theta }\in \varvec{\Theta }\}$ are defined with respect to a family of probability measures $\{ \mathbb {P}^{\varvec{\theta }} | \varvec{\theta }\in \varvec{\Theta }\}$ indexed by $\varvec{\Theta }$.

Furthermore, under $\mathbb {P}^{\varvec{\theta }}$, $\{ X_t \}_{t \in \mathbb {T}}$ follows the parametric nonlinear time series model:

3.7 $$\begin{aligned} X_t= & {} f (X_{t-1}, X_{t-2}, \ldots , X_{t-p}) + \mu g (X_{t-1}, X_{t-2}, \ldots , X_{t - q}) \nonumber \\&+\, \sigma g (X_{t-1}, X_{t-2}, \ldots , X_{t - q}) \epsilon _t (\varvec{\theta }). \end{aligned}$$

It may be noted that $\{ X_t \}_{t \in \mathbb {T}}$ follows the reference model in Eq. (3.1) under the reference measure $\mathbb {P}$. Under the reference model in Eq. (3.1), the conditional mean and volatility are given by the functions *f* and *g*, respectively. However, $\{ X_t \}_{t \in \mathbb {T}}$ follows an alternative model in Eq. (3.7) under the new probability measure $\mathbb {P}^{\varvec{\theta }}$ defined by Eq. (3.5), for each $\varvec{\theta }\in \varvec{\Theta }$. Under the alternative model in Eq. (3.7), the conditional mean and volatility are given by the functions $f + \mu g$ and $\sigma g$, respectively. That is, the effects of changing the probability measures from $\mathbb {P}$ to $\mathbb {P}^{\varvec{\theta }}$ are to (1) perturb the conditional mean function *f* by $\mu$ in the “direction” of the conditional volatility function *g* and (2) scale the conditional volatility function *g* by $\sigma$. When $\mu = 0$ and $\sigma = 1$, (i.e. there are no drift and volatility misspecifications), the alternative model in Eq. (3.7) coincides with the reference model in Eq. (3.1). Recall that $(\mu , \sigma )$ are the “uncertain” parameters for drift and volatility misspecifications since the misspecifications may not be known in practice. Consequently, it is uncertain which alternative model may be used. Instead of using a single alternative model, again, a family of alternative models may be used. Recall that the focus in the second stage is prediction. Consequently, a family of alternative models for prediction is specified. With the “uncertain” parameters $\varvec{\theta }:= (\mu , \sigma )$ varying in $\varvec{\Theta }:= [\mu ^-, \mu ^+] \times [\sigma ^-, \sigma ^+]$, a family of alternative models in the form of Eq. (3.7) for prediction is defined with respect to the family of probability measures $\{ \mathbb {P}^{\varvec{\theta }} | \varvec{\theta }\in \varvec{\Theta }\}$. That is, for each $\varvec{\theta }\in \varvec{\Theta }$, an alternative model under the probability measure $\mathbb {P}^{\varvec{\theta }}$ has the conditional mean function $f + \mu g$, the conditional volatility function $\sigma g$ and the sequence of i.i.d. standard normal random shocks $\epsilon (\varvec{\theta })$. It may be noted that for each $\varvec{\theta }\in \varvec{\Theta }$, the probability measure $\mathbb {P}^{\varvec{\theta }}$ defined by the discrete-time Girsanov’s transform in Eq. (3.5) is equivalent to the reference probability measure $\mathbb {P}$.

To incorporate the family of alternative models in prediction, (conditional) nonlinear expectations are considered here. For example, similar to the “worst-case” scenario approach to model uncertainty in economic decision-making, one may wish to make a conservative prediction by considering the “worst-case” scenario over the family of alternative models, say the “worst-case” scenario prediction. This may be achieved by considering a pair of conditional nonlinear expectations, namely a conditional sublinear expectation and a conditional superlinear expectation. Specifically, suppose that one wishes to predict a random variable *Y* given the information described by a sub-$\sigma$-field $\mathcal{H}$ of $\mathcal{F}$. Then a conditional sublinear expectation and a conditional superlinear expectation for *Y* given $\mathcal{H}$ with respect to $\{ \mathbb {P}^{\varvec{\theta }} | \varvec{\theta }\in \varvec{\Theta }\}$ defining the family of alternative models may be used. The conditional sublinear expectation and the conditional superlinear expectation for *Y* given $\mathcal{H}$ with respect to $\{ \mathbb {P}^{\varvec{\theta }} | \varvec{\theta }\in \varvec{\Theta }\}$ are, respectively, defined by:

3.8 $$\begin{aligned} \mathbb {E}^{S}_{\varvec{\Theta }} [Y | \mathcal{H} ] := \text{ ess }-\sup _{\varvec{\theta }\in \varvec{\Theta }} {\text{ E }}^{\varvec{\theta }} [Y | \mathcal{H} ], \end{aligned}$$

and

3.9 $$\begin{aligned} \mathbb {E}^{I}_{\varvec{\Theta }} [Y | \mathcal{H} ] := \text{ ess }-\inf _{\varvec{\theta }\in \varvec{\Theta }} {\text{ E }}^{\varvec{\theta }} [Y | \mathcal{H} ], \end{aligned}$$

where ${\text{ E }}^{\varvec{\theta }} [\cdot | \mathcal{H} ]$ is the conditional expectation given $\mathcal{H}$ under $\mathbb {P}^{\varvec{\theta }}$. $\text{ ess }-\sup$ and $\text{ ess }-\inf$ are the essential supremum and the essential infimum, respectively. The essential supremum and the essential infimum are taken with respect to the “uncertain’ parameters $\varvec{\theta }:= (\mu , \sigma )$ varying in the product intervals $\varvec{\Theta }$. Consequently, the family of alternative models defined by $\{ \mathbb {P}^{\varvec{\theta }} | \varvec{\theta }\in \varvec{\Theta }\}$ is incorporated in the definitions of the conditional sublinear expectation in Eq. (3.8) and the conditional superlinear expectation in Eq. (3.9). From the technical perspective, the essential supremum and the essential infimum are used since the conditional expectation ${\text{ E }}^{\varvec{\theta }} [Y | \mathcal{H} ]$ is a random variable, for each $\varvec{\theta }\in \varvec{\Theta }$. Also, to simplify the discussion, it is supposed that the random variable *Y* is integrable with respect to $\mathbb {P}^{\varvec{\theta }}$, (i.e. $\text{ E}^{\varvec{\theta }} [| Y |] < \infty$, where $\text{ E}^{\varvec{\theta }} [\cdot ]$ is the expectation under $\mathbb {P}^{\varvec{\theta }}$), for each $\varvec{\theta }\in \varvec{\Theta }$.

Depending on the purposes of applications, either the conditional sublinear expectation or the conditional superlinear expectation may be used as the “worst-case” scenario prediction. Specifically, if one wishes to predict the future profit (loss) described by the random variable *Y* based on the current and past information described by $\mathcal{H}$, then the conditional superlinear expectation (the conditional sublinear expectation) may be used as the “worst-case” scenario prediction. Instead of making a point prediction, one may also make an interval prediction based on a pair of conditional superlinear and sublinear expectations, where the former and the latter give the lower and upper limits of the interval prediction, respectively.

Whether one wishes to use the conditional nonlinear (sublinear and/or superlinear) expectations for making a point prediction or an interval prediction, a key issue to be addressed is to determine the upper limits, say $\mu ^+, \sigma ^+$, and the lower limits, say $\mu ^-, \sigma ^-$, in the product intervals $\varvec{\Theta }$. In the next section, concepts in Bayesian statistics will be used to inform the determination of the upper and lower limits in $\varvec{\Theta }$. Specifically, Bayesian credible intervals are used to determine the upper and lower limits in $\varvec{\Theta }$ to which certain probability levels can be assigned to indicate the degree of credibility that the family of alternative models defined by $\{ \mathbb {P}^{\varvec{\theta }} | \varvec{\theta }\in \varvec{\Theta }\}$ may have. The Bayesian credible intervals also incorporate both a model user’s subjective view (or expert opinion) and the objective data in determining the upper and lower limits in $\varvec{\Theta }$. The Bayesian nonlinear expectations for prediction in the second stage are then defined by using the conditional nonlinear expectations in Eq. (3.8) and Eq. (3.9) with the upper and lower limits in $\varvec{\Theta }$ determined by the Bayesian credible intervals as well as with the given information $\mathcal{H}$ specified by a $\sigma$-field generated by the time series observations.

### A product process

Though the reference model in Eq. (3.1) (i.e. a nonlinear autoregressive model with conditional heteroscedasticity) includes the GARCH model, it does not include a SV model. This is because the SV model involves two random shocks per unit of time while the reference model in Eq. (3.1) involves one random shock per unit of time. To include the SV model, a product process is needed. As noted in Sherphard (2005), the product process was introduced in an unpublished paper of Rosenberg (1972) and was studied in Taylor (1980, 1982). It was pointed out in Taylor (1982) that the product process is not included in some nonlinear time series models such as the bilinear model in Granger and Anderson (1978) and the state-dependent model in Priestley (1980) for the product process involves two random shocks per unit of time.

A reference model which includes the first model in Sect. 3.1 and a product process is now specified. Recall from Sect. 3.1 that $\{ \epsilon _t \}_{t \in \mathbb {T}}$ is a sequence of i.i.d. standard normal random variables under the reference probability measure $\mathbb {P}$. Let $\{ \eta _t \}_{t \in \mathbb {T}}$ be another sequence of i.i.d. standard normal random variables under $\mathbb {P}$. Assume that the two sequences $\{ \epsilon _t \}_{t \in \mathbb {T}}$ and $\{ \eta _t \}_{t \in \mathbb {T}}$ are independent under $\mathbb {P}$. It is supposed that under $\mathbb {P}$, $\{ X_t \}_{t \in \mathbb {T}}$ follows the reference parametric nonlinear time series model:

3.10 $$\begin{aligned} X_t= & {} f (X_{t-1}, X_{t-2}, \ldots , X_{t-p}) + h (X_{t-1}, X_{t-2}, \ldots , X_{t-q}, V_t) \epsilon _t, \nonumber \\ V_t= & {} f_v (V_{t-1}, V_{t-2}, \ldots , V_{t-p_v}) + h_v (V_{t-1}, V_{t-2}, \ldots , V_{t-q_v}) \eta _t, \end{aligned}$$

where $f : \Re ^p \rightarrow \Re$, $h : \Re ^{q+1} \rightarrow \Re$, $f_v : \Re ^{p_v} \rightarrow \Re$ and $h_v : \Re ^{q_v} \rightarrow \Re$ are given measurable functions; $h > 0$ and $h_v > 0$; *p*, *q*, $p_v$ and $q_v$ are positive integers. $\{ V_t \}_{t \in \mathbb {T}}$ is a latent process, which is a stochastic process defined on $(\Omega , \mathcal{F}, \mathbb {P})$. Defining the time series process $\{ X_t \}_{t \in \mathbb {T}}$ and the latent process $\{ V_t \}_{t \in \mathbb {T}}$ on the same probability space $(\Omega , \mathcal{F}, \mathbb {P})$ may avoid the complexity of introducing a product probability space. It is assumed that the initial values $\{ V_{- \max (p_v, q_v)}, V_{- \max (p_v, q_v) + 1}, \ldots , V_0 \}$ are also random variables defined on $(\Omega , \mathcal{F}, \mathbb {P})$. It may be noted that there are two random shocks per unit of time in the reference model in Eq. (3.10). Specifically, at each time $t \in \mathbb {T}$, there are two random shocks $\epsilon _t$ and $\eta _t$, where $\epsilon _t$ is the random shock driving the time series process $\{ X_t \}_{t \in \mathbb {T}}$ and $\eta _t$ is the random shock driving the latent process $\{ V_t \}_{t \in \mathbb {T}}$. Consequently, the reference model in Eq. (3.10) includes a product process, which in turn includes the SV model. Also, if the volatility function *h* in the first equation of Eq. (3.10) becomes the volatility function *g* in Eq. (3.1), then the reference model in Eq. (3.10), (i.e. the second model), coincides with the reference model in Eq. (3.1), (i.e. the first model).

Suppose that

3.11 $$\begin{aligned}&f (X_{t-1}, X_{t-2}, \ldots , X_{t-p}) = 0, \quad h (X_{t-1}, X_{t-2}, \ldots , X_{t-q}, V_t) = \exp (V_t / 2), \nonumber \\&f_v (V_{t-1}, V_{t-2}, \ldots , V_{t-p_v}) = \alpha _v + \delta _v V_{t-1}, \quad h_v (V_{t-1}, V_{t-2}, \ldots , V_{t-q_v}) = \sigma _v. \end{aligned}$$

Then the reference model in Eq. (3.10) becomes:

3.12 $$\begin{aligned} X_t= & {} \exp (V_t / 2) \epsilon _t, \nonumber \\ V_t= & {} \alpha _v + \delta _v V_{t-1} + \sigma _v \eta _t. \end{aligned}$$

This is the discrete-time SV model presented in Jacquier et al. (2002). The discrete-time SV model, which is also called a lognormal autoregressive model for a latent volatility process, was first proposed by Taylor (1982). For Bitcoin applications to be presented in Sect. 6, the discrete-time SV model in Eq. (3.12) will be used.

After specifying the reference model in Eq. (3.10) in the first stage, the second stage is considered. Again, a discrete-time Girsanov’s transform is used to construct a family of probability measures equivalent to the reference measure $\mathbb {P}$. The family of probability measures is used to define Gaussian “uncertain” noises and a family of alternative models from the reference model in Eq. (3.10). The constructions and derivations are presented in Online Appendix B.

## Bayesian nonlinear expectations

The second stage of the proposed two-stage approach is concerned with prediction, which is an ultimate goal of time series modelling. If an estimated reference model from the first stage is used for prediction, there are two concerns. Firstly, as discussed in Sect. 3, there may be model misspecifications or uncertainties in the conditional mean and volatility of the reference model. Indeed, the “true” underlying data generating process, if exists, is often unknown to model users in practice. Consequently, it appears that model misspecifications or uncertainties are ubiquitous. Secondly, the future may not follow from the present and the past. Consequently, there may be a concern that the future developments of a time series process may not follow what are predicted from the reference model estimated from the present and past data. To address these concerns, a family of alternative models is used to predict the future developments of the time series process in the second stage. Specifically, the discrete-time Bayesian nonlinear expectations incorporating a family of alternative models for prediction are constructed in this section. To construct the Bayesian nonlinear expectations, the first step is to evaluate the residuals of an estimated reference model. The residuals may be thought of as proxies/estimates for the random errors in the reference model which in turns contain information that is left over due to model misspecifications in the conditional mean and volatility of the reference model. Then a Bayesian model for the random errors in the reference model is constructed by treating the “uncertain” parameters for drift and volatility misspecifications as random variables. Using conjugate priors for the “uncertain” parameters and the residuals of the estimated reference model as proxies/estimates for the random errors, closed-form Bayesian credible intervals for the “uncertain” parameters are obtained. The closed-form Bayesian credible intervals, specifically their lower and upper limits, are then used to determine the lower and upper limits of the product intervals indexing the family of probability measures which defines the family of alternative models to be incorporated in prediction. Effectively, these alternative models are introduced by extracting information that is left over due to model misspecifications in the conditional mean and volatility of the reference model via Bayesian credible intervals. The Bayesian nonlinear expectations are given by the conditional nonlinear expectations with the lower and upper limits of the product intervals determined by the Bayesian credible intervals.

Bayesian statistics, as it may be quite well-known, is an important approach to model uncertainty. Indeed, Bayesian statistics has a solid foundation in decision theory under uncertainty as established in, for example, Bernardo and Smith (2000). As far as prediction is concerned, the Bayesian averaging approach has been adopted to incorporate model uncertainty in evaluating Bayesian predictive distributions. The basic idea of the Bayesian averaging approach is to evaluate the predictive distributions by averaging out full posteriors of the unknown parameters. If the model has many unknown parameters, the evaluation of the predictive distributions using the Bayesian averaging approach will involve computing high-dimensional integrals. Also, if the parametric form of the model is complicated, closed-form solutions to the posteriors may be very difficult, if not impossible, to obtain using conjugate priors. In this case, numerical simulations based on, for example, Markov Chain Monte Carlo (MCMC) methods are required for computing the full posteriors. It appears that the Bayesian averaging approach may be related to the smooth ambiguity approach for decision-making with model uncertainty proposed in Klibanoff et al. (2005), where Bayesian averaging was used in constructing the criterion for preferences. The Bayesian nonlinear expectations to be introduced in this section provide an alternative way to incorporate model uncertainties or misspecifications in prediction. Like the conditional nonlinear expectations as discussed in Sect. 3.1, the Bayesian nonlinear expectations can be used to provide a conservative prediction by considering the “worst-case” scenario over the family of alternative models, say the “worst-case” scenario prediction. It may be thought that the Bayesian nonlinear expectations combine concepts in Bayesian statistics and the “worst-case” scenario approach to model uncertainty for making predictions. Using the closed-form Bayesian credible intervals based on conjugate priors, the Bayesian nonlinear expectations can be easily computed and provide a convenient way to make predictions. Furthermore, the use of the Bayesian credible intervals allows probability levels to be assigned with the objective of indicating credibility of predictions made by Bayesian nonlinear expectations. As pointed out by Hansen (2013), Page 431 therein, under the smooth ambiguity approach, ambiguity was identified as a characteristic of subjective beliefs of a model user and a Bayesian prior was assigned to alternative models. This may also apply to both the Bayesian averaging approach and the Bayesian nonlinear expectations approach. Indeed, both the Bayesian averaging approach and the Bayesian nonlinear expectations approach incorporate a model user’s subjective view (or expert opinion) and the objective data in making predictions. The model user’s subjective view (or expert opinion) may be used to adjust predictions based solely on the present and the past data in case the future may not follow the present and the past.

In the sequel, the Bayesian credible intervals and the Bayesian nonlinear expectations corresponding to the first model in Sect. 3.1 will be constructed. The construction of the Bayesian credible intervals and the Bayesian nonlinear expectations corresponding to the second model in Sect. 3.2 follows similarly and is presented in Online Appendix C. Some formulas and notations in Bernardo and Smith (2000), (see, for example, Pages 121 and 440 therein), will be used. For an excellent account of Bayesian statistics, one may refer to, for example, Box and Tiao (1992), Bernardo and Smith (2000) and Lee (2012). In Online Appendix D, a (potential) fusion between Bayesian nonlinear expectations and the Bayesian shrinkage and regularization techniques for estimation and variable selection is discussed using the proposed two-stage approach. The variable selection is an important topic in econometrics, statistics and machine learning. As far as the sparsity of a high-dimensional regression model is concerned, the Bayesian shrinkage and regularization techniques in the Bayesian model selection literature are the key techniques for estimation and variable selection, (see, for example, Carvalho et al. 2009; Polson and Scott 2010; Bhadra et al. 2019 and the relevant references therein). It may be of interest to explore the (potential) link between the Bayesian shrinkage and regularization techniques and the Bayesian nonlinear expectations.

The Bayesian nonlinear expectations corresponding to the first model in Sect. 3.1 are now constructed. From Eq. (3.6), for each $\varvec{\theta }\in \varvec{\Theta }$,

4.1 $$\begin{aligned} \epsilon _t = \mu + \sigma \epsilon _t (\varvec{\theta }), \end{aligned}$$

where $(\mu , \sigma )$ are the “uncertain” parameters for the drift and volatility misspecifications in the reference model in Eq. (3.1). In Bayesian statistics, $\mu$ and $\sigma$ are assumed to be random variables, and prior distributions for $\mu$ and $\sigma$ are assigned. Recall from Sect. 3.1 that $\{ \epsilon _t (\varvec{\theta }) \}_{t \in \mathbb {T}}$ is a sequence of i.i.d. standard normal random variables under the probability measure $\mathbb {P}^{\varvec{\theta }}$, for each $\varvec{\theta }\in \varvec{\Theta }$. Then from Eq. (4.1), under $\mathbb {P}^{\varvec{\theta }}$, given $(\mu , \sigma )$, $\{ \epsilon _t \}_{t \in \mathbb {T}}$ is a sequence of conditionally i.i.d. normal random variables with mean $\mu$ and standard deviation $\sigma$. That is, under $\mathbb {P}^{\varvec{\theta }}$, $\epsilon _1, \epsilon _2, \ldots , \epsilon _T | (\mu , \sigma ) \overset{i.i.d.}{\sim } N(\mu , \sigma ^2)$. It may be noted that under the reference probability measure $\mathbb {P}$, $\epsilon _1, \epsilon _2, \ldots , \epsilon _T \overset{i.i.d.}{\sim } N(0, 1)$. Here the precision $\lambda$, where $\lambda = \frac{1}{\sigma ^2}$, is considered, and a prior distribution is assigned for $\lambda$ instead of $\sigma$. Again, if $\lambda = 1$, then there is no volatility misspecification in a reference model. If $\lambda > 1$, (i.e. $\sigma < 1$), then the volatility specification in the reference model in Eq. (3.1) may be too high. If $\lambda < 1$, (i.e. $\sigma > 1$), then the volatility specification in the reference model in Eq. (3.1) may be too low. Consequently, either $\lambda$ or $\sigma$ may be used to describe volatility misspecifications in the reference model in Eq. (3.1).

To assign prior distributions for $(\mu , \lambda )$, a normal-gamma prior distribution is considered. Specifically,

4.2 $$\begin{aligned} \mu | \lambda \sim N \bigg (\mu _0, \frac{1}{t_0 \lambda } \bigg ), \quad \lambda \sim Ga (\alpha , \beta ), \end{aligned}$$

where $N (\mu _0, \frac{1}{t_0 \lambda })$ is a normal distribution with mean $\mu _0$ and variance $\frac{1}{t_0 \lambda }$, and $Ga (\alpha , \beta )$ is a Gamma distribution with shape parameter $\alpha$ and rate parameter $\beta$. Consequently, the prior mean of $\mu$ is $\mu _0$ and the prior variance of $\mu | \lambda$ is $\frac{1}{t_0 \lambda }$. The prior mean $\mu _0$ may be thought of as a prior estimate for $\mu$, and the prior precision $t_0 \lambda$ may indicate the strength of belief on the prior estimate $\mu _0$. Say, for a fixed value of $\lambda$, a larger (smaller) prior parameter $t_0$ may express a stronger (weaker) belief on the prior estimate $\mu _0$. Furthermore, the prior mean and variance of $\lambda$ are $\frac{\alpha }{\beta }$ and $\frac{\alpha }{\beta ^2}$, respectively. A larger (smaller) prior variance $\frac{\alpha }{\beta ^2}$ may express a weaker (stronger) belief on the prior estimate $\frac{\alpha }{\beta }$ for $\lambda$. Recall that the random variables $\mu$ and $\lambda$ describe misspecifications in the drift and volatility in the reference model in Eq. (3.1). Consequently, when $\lambda$ becomes smaller, (i.e. $\lambda < 1$), for a fixed prior parameter $t_0$, the belief on the prior estimate $\mu _0$ becomes weaker, (i.e. $t_0 \lambda$ becomes smaller). To explain the intuition behind this, when $\lambda$ becomes smaller, the volatility specification in the reference model in Eq. (3.1) may be too low. In this case, the conditional mean specification in the reference model may dominate, in a relative sense, its volatility specification in prediction. Consequently, misspecifications in the conditional mean may have a more pronounced impact on the prediction than misspecifications in the volatility. To be cautious, one may express a weaker belief on the prior estimate $\mu _0$ for misspecifications in the conditional mean. Likewise, when $\lambda$ becomes larger, (i.e. $\lambda > 1$), for a fixed prior parameter $t_0$, the belief on the prior estimate $\mu _0$ becomes stronger, (i.e. $t_0 \lambda$ becomes larger). The intuition behind this can be explained similarly. The prior parameters $\mu _0$, $t_0$, $\alpha$ and $\beta$, which are also called the hyperparameters, are chosen by a model user according to the model user’s prior/subjective belief (or expert opinion) on the drift and volatility misspecifications. They will then be combined with the data on the residuals of the reference model to compute the posterior distributions for the drift and volatility misspecifications rationally by the Bayes’ formula.

Let $\{ X_1, X_2, \ldots , X_n \}$ be a sequence of observations of a time series process, where *n* is the number of observations. Write $\mathbb {N}$ for the indices of the observations, where $\mathbb {N} := \{1, 2, \ldots , n \}$. After estimating the reference model in Eq. (3.1) using the observations $\{ X_1, X_2, \ldots , X_n \}$ in the first stage, which will be done by the maximum likelihood estimation (MLE) as will be discussed in Sect. 5.1, the residuals of the reference model $\{ e_t \}_{t \in \mathbb {N}}$ can be computed as follows:

4.3 $$\begin{aligned} e_t := \frac{X_t - {\hat{f}} (X_{t-1}, X_{t-2}, \ldots , X_{t-p})}{{\hat{g}} (X_{t-1}, X_{t-2}, \ldots , X_{t-q})}, \end{aligned}$$

where ${\hat{f}}$ and ${\hat{g}}$ are the estimates for the conditional mean *f* and the volatility *g*, respectively. For each $t \in \mathbb {N}$, the residual $e_t$ is a proxy (or an estimate) for the random error $\epsilon _t$. Recall that under $\mathbb {P}^{\varvec{\theta }}$, $\epsilon _1, \epsilon _2, \ldots , \epsilon _n | (\mu , \sigma ) \overset{i.i.d.}{\sim } N(\mu , \sigma ^2)$. It is also assumed that as an approximation, under $\mathbb {P}^{\varvec{\theta }}$, $e_1, e_2, \ldots , e_n | (\mu , \sigma ) \overset{i.i.d.}{\sim } N(\mu , \sigma ^2)$.

Suppose that the observations $\mathbf{e} (n) := (e_1, e_2, \ldots , e_n)$ are now given. Let

4.4 $$\begin{aligned} \beta _n := \beta + \frac{1}{2} n s^2 + \frac{t_0 n (\mu _0 - {\bar{e}})^2 }{2 (t_0 + n)}, \end{aligned}$$

where ${\bar{e}} = \frac{1}{n} \sum ^{n}_{t = 1} e_t$, (i.e. the sample mean of $\mathbf{e} (n)$), and $s^2 = \frac{1}{n} \sum ^{n}_{t = 1} (e_t - {\bar{e}})^2$, (i.e. the sample variance of $\mathbf{e} (n)$). Then it is known (see Bernardo and Smith 2000, Page 440) that by the Bayes’ formula, the posterior distribution of $\lambda$ given $\mathbf{e} (n)$ is:

4.5 $$\begin{aligned} \lambda | \mathbf{e} (n) \sim Ga \bigg (\alpha + \frac{n}{2}, \beta _n \bigg ), \end{aligned}$$

where $\alpha + \frac{n}{2}$ is the posterior shape parameter of $\lambda$ given $\mathbf{e} (n)$, and $\beta _n$, which is given by Eq. (4.4), is the posterior rate parameter of $\lambda$ given $\mathbf{e} (n)$.

Let

4.6 $$\begin{aligned} \mu _n := \frac{t_0 \mu _0 + n {\bar{e}} }{t_0 + n}, \end{aligned}$$

and

4.7 $$\begin{aligned} \sigma _n := \sqrt{\frac{\beta _n}{\alpha + \frac{1}{2} n}}, \end{aligned}$$

where $\beta _n$ is given by Eq. (4.4). Then, it is also known (see Bernardo and Smith 2000, Page 440) that by the Bayes’ formula, the posterior distribution of $\mu$ given $\mathbf{e} (n)$ is:

4.8 $$\begin{aligned} \mu | \mathbf{e} (n) \sim St \bigg (\mu _n, \frac{\sigma ^2_n}{(t_0 + n)}, 2 \alpha + n \bigg ), \end{aligned}$$

Here *St*(*a*, *b*, *c*) is a Student’s-*t* distribution with a location parameter *a*, a scale parameter $\sqrt{b}$, (or the precision $b^{-1}$), and the degree of freedom *c*; $\mu _n$ in Eq. (4.6) is the posterior location parameter for $\mu$ given $\mathbf{e} (n)$. $\frac{\sigma _n}{\sqrt{t_0 + n}}$ is the posterior scale parameter of $\mu$ given $\mathbf{e} (n)$, where $\sigma _n$ is given by Eq. (4.7).

Then a $100 (1 - \gamma _1) \%$ Bayesian credible interval for $\mu$ given $\mathbf{e} (n)$ is $(L_{\mu } (\gamma _1), U_{\mu } (\gamma _1))$ such that the lower limit $L_{\mu } (\gamma _1)$ and the upper limit $U_{\mu } (\gamma _1)$ are, respectively, given by:

4.9 $$\begin{aligned} L_{\mu } (\gamma _1) = \mu _n - t_{2 \alpha + n} \bigg (\frac{\gamma _1}{2} \bigg ) \frac{\sigma _n}{\sqrt{n + t_0}}, \end{aligned}$$

and

4.10 $$\begin{aligned} U_{\mu } (\gamma _1) = \mu _n + t_{2 \alpha + n} \bigg (\frac{\gamma _1}{2} \bigg ) \frac{\sigma _n}{\sqrt{n + t_0}}. \end{aligned}$$

Here $t_{2 \alpha + n} (\frac{\gamma _1}{2} )$ is the critical value of a Student’s-*t* distribution with degree of freedom $2 \alpha + n$ such that a Student’s-*t* random variable with the same degree of freedom is less than or equal to $t_{2 \alpha + n} (\frac{\gamma _1}{2} )$ with probability $1 - \frac{\gamma _1}{2}$. When the sample size *n* becomes large as in the majority of time series data encountered in practice, the Student’s-*t* distribution with degree of freedom $2 \alpha + n$ tends to a normal distribution. In this case, the normal distribution can be used to construct the Bayesian credible interval for $\mu$ given $\mathbf{e} (n)$. However, for the sake of generality, the Student’s-*t* distribution is considered here since it can also capture the situation of short time series data.

Similarly, a $100 (1 - \gamma _2) \%$ Bayesian credible interval for $\lambda$ given $\mathbf{e} (n)$ is $(L_{\lambda } (\gamma _2), U_{\lambda } (\gamma _2))$ such that the lower limit $L_{\lambda } (\gamma _2)$ and the upper limit $U_{\lambda } (\gamma _2)$ are, respectively, given by:

4.11 $$\begin{aligned} L_{\lambda } (\gamma _2) = \Gamma _{\alpha + \frac{n}{2}, \beta _n} \bigg (1 - \frac{\gamma _2}{2} \bigg ), \end{aligned}$$

and

4.12 $$\begin{aligned} U_{\lambda } (\gamma _2) = \Gamma _{\alpha + \frac{n}{2}, \beta _n} \bigg (\frac{\gamma _2}{2} \bigg ), \end{aligned}$$

where $\Gamma _{\alpha + \frac{n}{2}, \beta _n} (\gamma )$ is the critical value of a Gamma distribution with a shape parameter $\alpha + \frac{n}{2}$ and a rate parameter $\beta _n$ such that a random variable following this distribution is less than or equal to $\Gamma _{\alpha + \frac{n}{2}, \beta _n} (\gamma )$ with probability $1 - \gamma$.

We now take:

4.13 $$\begin{aligned} \mu ^- = \mu ^- (\gamma _1) = L_{\mu } (\gamma _1), \quad \mu ^+ = \mu ^+ (\gamma _1) = U_{\mu } (\gamma _1), \end{aligned}$$

and

4.14 $$\begin{aligned} \sigma ^- = \sigma ^- (\gamma _2) = \sqrt{\frac{1}{U_{\lambda } (\gamma _2) }}, \quad \sigma ^+ = \sigma ^+ (\gamma _2) = \sqrt{\frac{1}{L_{\lambda } (\gamma _2) }}. \end{aligned}$$

Consequently, the product intervals $\varvec{\Theta }$ in Eq. (3.2) are taken as the following product intervals $\varvec{\Theta }_{\gamma _1, \gamma _2} (\mathbf{e} (n))$ with probability levels $\gamma _1$ and $\gamma _2$:

4.15 $$\begin{aligned} \varvec{\Theta }_{\gamma _1, \gamma _2} (\mathbf{e} (n)) := [ \mu ^- (\gamma _1), \mu ^+ (\gamma _1)] \times [\sigma ^- (\gamma _2), \sigma ^+ (\gamma _2)], \end{aligned}$$

and a family of alternative models is defined by the family of probability measures $\{ \mathbb {P} (\varvec{\theta }) | \varvec{\theta }\in \varvec{\Theta }_{\gamma _1, \gamma _2}(\mathbf{e} (n)) \}$ indexed by $\varvec{\Theta }_{\gamma _1, \gamma _2} (\mathbf{e} (n))$. To emphasize that the product intervals in Eq. (4.15) depend on the observations $\mathbf{e} (n)$ of the residuals, the notation $\varvec{\Theta }_{\gamma _1, \gamma _2} (\mathbf{e} (n))$ is used.

It may be noted that the Bayesian credible intervals are used in the second stage which is concerned with prediction, and they are not considered in the first stage which is concerned with the estimation of a reference model. Intuitively, the Bayesian credible intervals combine a model user’s prior/subjective belief (or expert opinion) via choosing the prior parameters (or hyperparameters) and information about the residuals $\mathbf{e} (n)$ to generate the family of alternative models with the objective of incorporating drift and volatility misspecifications described by the “uncertain” parameters $(\mu , \sigma )$. For example, the 90% Bayesian credible intervals for $\mu$ and $\sigma$, which can be constructed based on the 5th and 95th percentiles of the posterior distributions for $\mu$ and $\sigma$, may be used to generate the family of alternative models which have the degree of credibility for capturing the drift and volatility misspecifications described by the 90% probability level. In line with the Bayesian interpretation, the 90% probability level may be thought of as a subjective probability level.

A Bayesian (posterior) sublinear expectation for an integrable random variable *Y* given the $\sigma$-field $\mathcal{F}^X_n$ generated by the time series observations $\{X_1, X_2, \ldots , X_n \}$ with respect to $\{ \mathbb {P} (\varvec{\theta }) | \varvec{\theta }\in \varvec{\Theta }_{\gamma _1, \gamma _2}(\mathbf{e} (n)) \}$ is constructed as follows:4.16Similarly, a Bayesian (posterior) superlinear expectation for an integrable random variable *Y* given $\mathcal{F}^X_n$ with respect to $\{ \mathbb {P} (\varvec{\theta }) | \varvec{\theta }\in \varvec{\Theta }_{\gamma _1, \gamma _2}(\mathbf{e} (n)) \}$ is defined as:4.17

## Estimation, forecasting and risk evaluation

The estimation, forecasting and risk evaluation procedures are described along the line of the two-stage approach. At the first stage, the estimation of the reference model in either Eq. (3.1) or Eq. (3.10) is considered. At the second stage, to incorporate the effects of drift and volatility misspecifications or uncertainties, in prediction, the Bayesian nonlinear expectations are used for forecasting and risk evaluation. For illustration, one-step-ahead forecasts for time series observations are adopted, and one-step-ahead predictions for VaR and ES are considered for risk evaluation.

### Estimation

The focus of this paper is parametric nonlinear time series modelling. It is supposed that the parametric forms in the reference models in Eq. (3.1) or Eq. (3.10) are given a priori. Specifically, the parametric forms of the drift function *f* and the volatility function *g* are given in the reference model in Eq. (3.1), and the parametric forms of the drift functions *f*, $f_v$ and the volatility functions *h* and $h_v$ are given in the reference model in Eq. (3.10). In these cases, the estimation of the two reference models becomes the estimation of the parameters in the two reference models. Since the reference model in Eq. (3.1) does not involve latent processes, once the parametric forms of the drift and volatility functions are given, the maximum likelihood estimation (MLE) may be used to estimate its parameters. Since the reference model in Eq. (3.10) involves a latent process, once the parametric forms of the drift and volatility functions are given, one may first need to estimate the latent process using filtering techniques (or nonlinear filtering techniques in the case when either the observation process or the latent process is nonlinear) and then use the quasi-MLE (QMLE) to estimate the parameters. Indeed, one may also use a fully Bayesian approach coupled with Markov Chain Monte Carlo (MCMC) to estimate the two reference models. The fully Bayesian approach coupled with MCMC gives full posterior distributions for unknown parameters, while the MLE and the QMLE give point estimates. While the fully Bayesian approach coupled with MCMC is flexible, the point estimates for the unknown parameters in a reference model are what we need in the first stage of the two-stage approach. Using these point estimates, the residuals of a reference model are computed, which are then used to construct the Bayesian credible intervals in the second stage. Of course, we may also use the point estimates from the posterior means or modes for the unknown parameters to compute the residuals of the reference model. However, the uses of the MLE and the QMLE for the estimation of the reference models may provide a clear illustration for the difference between the objectives of the first and second stages in the proposed approach.

Furthermore, the MLE and the QMLE with the Kalman filter are easy to implement for the SETAR model, the GARCH model and the SV model. The Bayesian MCMC estimation of these three models involves numerical simulations of full posterior distributions for the unknown parameters/latent variables, which could be more complicated. The three models will be estimated using real Bitcoin data in Sect. 6. Specifically, when the reference model is specified as either the SETAR model or the GARCH model, the MLE is used to estimate the model; when the reference model is specified as the SV model, the QMLE coupled with the Kalman filter are used to estimate the model. Indeed, the estimation of the SETAR model and the GARCH model can be done using the function “tar” in the R package “TSA” and the function “garch” in the R package “tseries”, respectively. The estimation of the SV model using the QMLE with the Kalman filter can be implemented quite conveniently using R following the filtering and estimation equations in Appendix B of Jacquier et al. (2002). The estimation of a SV model using the QMLE with the Kalman filter can also be implemented using Excel spreadsheets, (see Taylor 2005, Chapter 11 therein). Besides the QMLE with the Kalman filter, there are other methods to estimate the SV model. See, for example, Taylor (1982, 1986) for moment estimates, Melino and Turnbull (1990) and Jacquier et al. (2002) for the generalized method of moments, Jacquier et al. (2002) and Nakajima and Omori (2009) for a fully Bayesian MCMC approach, Kim et al. (1998) and Omori et al. (2007) for the likelihood-based approaches.

### Forecasting

An upper point forecast, a lower point forecast and an interval forecast for the daily percentage logarithmic return of a financial asset in the next time period given information about the returns up to and including the current time period are obtained under each of the reference models in Eq. (3.1) and Eq. (3.10). The upper point forecast and the lower point forecast are defined using a Bayesian posterior sublinear and superlinear expectations, respectively. Likewise, the lower and upper limits of the interval forecast are specified by a Bayesian posterior sublinear and superlinear expectations, respectively. The interval forecast considered here may entail a different interpretation from the prediction interval used in forecasting. Specifically, the former indicates model uncertainty while the latter indicates uncertainty due to the variability of a forecast. The distinction between the interval forecast considered here and a prediction interval may be illustrated by considering the distinction between uncertainty and risk in the classic work by Knight (1921). Specifically, the interval forecast considered here may be related to the notion of uncertainty while a prediction interval may be related to the concept of risk in the sense of Knight (1921).

As a comparison, a Bayesian risk-neutral forecast based on the posterior means of the “uncertain” parameters is also obtained under each of the reference models in Eq. (3.1) and Eq. (3.10). The derivations of the upper, lower and interval forecasts as well as the Bayesian risk-neutral forecasts under the two reference models in Eq. (3.1) and Eq. (3.10) are provided in Online Appendix E.

### Risk measures

Two tail-based risk metrics, namely value at risk (VaR) and expected shortfall (ES), are adopted here to evaluate risk. VaR has been a popular risk metric. However, it has been pointed out that VaR does not, in general, satisfy the sub-additivity property. Intuitively, this means that diversification of risk may be penalized if VaR is used as a risk metric. An alternative risk metric, namely ES, has been proposed in, for example, Artzner et al. (1999), which satisfies the sub-additivity property when the loss distribution is continuous and takes account of the tail risk beyond the VaR threshold. See, for example, Artzner et al. (1999) and Boyle et al. (2002) for related discussions.

Again, one-step-ahead forecasts are considered for illustration. This does not seem to be an unreasonable consideration for, in practice, risk metrics such as VaR are often evaluated or updated for each period, say one day. Since these estimates of the risk metrics are evaluated based on the Bayesian posterior distributions, they combine both expert opinion and objective market data when evaluating risks, and these risk metrics may be related to Bayesian coherent risk measures in, for example, Siu et al. (2001), and Bayesian Value at Risk in, for example, Siu et al. (2004).

A short position for one unit of a Bitcoin is considered for the computation of the estimates of the VaR and ES. This will be used in the real data applications to be presented in Sect. 6. A short position of Bitcoin was also considered for evaluating VaR and ES in Siu (2021). Firstly, the reference model in Eq. (3.1) is considered. Let $X_t$ be the loss random variable of the short position of the Bitcoin in the *t*th period. Since (percentage) logarithmic returns may be used to approximate percentage changes in the value of a risky portfolio, the loss random variable $X_t$ is equal to the logarithmic return in the *t*th-period, where $X_t$ is expressed as percentage. See, for example, Tsay (2013), Page 337.

Let ${\text{ V }aR}^{\varvec{\theta }}_{1-p} [X_{n+1} | \mathcal{F}^X_n ]$ and ${\text{ E }S}^{\varvec{\theta }}_{1-p} [X_{n+1} | \mathcal{F}^X_n ]$ be the VaR and ES of the short position of the Bitcoin in the $(n+1)^{st}$-period given $\mathcal{F}^X_n$ with confidence level $1 - p$ evaluated under the probability measure $\mathbb {P}^{\varvec{\theta }}$, respectively. For example, *p* could be 0.05. Then applying Eq. (7.2) on Page 332 and Eq. (7.6) on Page 335 in Tsay (2013) and using the notation introduced in Online Appendix E and Eq. (3.7), ${\text{ V }aR}^{\varvec{\theta }}_{1-p} [X_{n+1} | \mathcal{F}^X_n ]$ and ${\text{ E }S}^{\varvec{\theta }}_{1-p} [X_{n+1} | \mathcal{F}^X_n ]$ have the following analytical expressions:

5.1 $$\begin{aligned} {\text{ V }aR}^{\varvec{\theta }}_{1-p} [X_{n+1} | \mathcal{F}^X_n ] = {\hat{f}} + \mu {\hat{g}} + \sigma {\hat{g}} z_{1 - p}, \end{aligned}$$

and

5.2 $$\begin{aligned} {\text{ E }S}^{\varvec{\theta }}_{1-p} [X_{n+1} | \mathcal{F}^X_n ] = {\hat{f}} + \mu {\hat{g}} + \sigma {\hat{g}} \frac{ \phi (z_{1 - p})}{p}, \end{aligned}$$

where $z_{1-p}$ and $\phi (\cdot )$ denote the $(1-p)$th quantile and the pdf of a standard normal distribution *N*(0, 1), respectively.

The upper-point estimates for the VaR in Eq. (5.1) and the ES in Eq. (5.2) with respect to $\{ \mathbb {P}^{\varvec{\theta }} | \varvec{\theta }\in \varvec{\Theta }_{2 \gamma _1, 2 \gamma _2} (\mathbf{e} (n)) \}$ are, respectively, given by:

5.3 $$\begin{aligned} {\text{ U }VaR}_{1-p, \varvec{\Theta }_{2 \gamma _1, 2 \gamma _2}(\mathbf{e} (n)) } [X_{n+1} | \mathcal{F}^X_n ] = \text{ ess }-\sup _{\varvec{\theta }\in \varvec{\Theta }_{2 \gamma _1, 2 \gamma _2}(\mathbf{e} (n)) } {\text{ V }aR}^{\varvec{\theta }}_{1-p} [X_{n+1} | \mathcal{F}^X_n ],\nonumber \\ \end{aligned}$$

and

5.4 $$\begin{aligned} {\text{ U }ES}_{1-p, \varvec{\Theta }_{2 \gamma _1, 2 \gamma _2}(\mathbf{e} (n)) } [X_{n+1} | \mathcal{F}^X_n ] = \text{ ess }-\sup _{\varvec{\theta }\in \varvec{\Theta }_{2 \gamma _1, 2 \gamma _2}(\mathbf{e} (n)) } {\text{ E }S}^{\varvec{\theta }}_{1-p} [X_{n+1} | \mathcal{F}^X_n ].\nonumber \\ \end{aligned}$$

The lower-point estimates for the VaR in Eq. (5.1) and the ES in Eq. (5.2) with respect to $\{ \mathbb {P}^{\varvec{\theta }} | \varvec{\theta }\in \varvec{\Theta }_{2 \gamma _1, 2 \gamma _2} (\mathbf{e} (n)) \}$, denoted by ${\text{ L }VaR}_{1-p, \varvec{\Theta }_{2 \gamma _1, 2 \gamma _2}(\mathbf{e} (n)) } [X_{n+1} | \mathcal{F}^X_n ]$ and ${\text{ L }ES}_{1-p, \varvec{\Theta }_{2 \gamma _1, 2 \gamma _2}(\mathbf{e} (n)) } [X_{n+1} | \mathcal{F}^X_n ]$, are, respectively, defined by replacing “$\text{ ess }-\sup$” in Eq. (5.3) and Eq. (5.4) with the “$\text{ ess }-\inf$”.

An interval estimate for the VaR in Eq. (5.1) with respect to $\{ \mathbb {P}^{\varvec{\theta }} | \varvec{\theta }\in \varvec{\Theta }_{\gamma _1, \gamma _2} (\mathbf{e} (n)) \}$ is given by:

5.5 $$\begin{aligned} ({\text{ L }VaR}_{1-p, \varvec{\Theta }_{\gamma _1, \gamma _2}(\mathbf{e} (n)) } [X_{n+1} | \mathcal{F}^X_n ], {\text{ U }VaR}_{1-p, \varvec{\Theta }_{\gamma _1, \gamma _2}(\mathbf{e} (n)) } [X_{n+1} | \mathcal{F}^X_n ]). \end{aligned}$$

Similarly, an interval estimate for the ES in Eq. (5.2) with respect to $\{ \mathbb {P}^{\varvec{\theta }} | \varvec{\theta }\in \varvec{\Theta }_{\gamma _1, \gamma _2} (\mathbf{e} (n)) \}$ is evaluated. The Bayesian risk-neutral estimate for the VaR is given by:

5.6 $$\begin{aligned} {\text{ B }RNVaR}_{1-p} [X_{n+1} | \mathcal{F}^X_n ] = {\hat{f}} + \mu _n {\hat{g}} + \sigma _n {\hat{g}} z_{1 - p}, \end{aligned}$$

where $\mu _n$ and $\sigma _n$ are given by Eq. (4.6) and Eq. (4.7), respectively. Similarly, the Bayesian risk-neutral estimate for the ES is evaluated.

Now the reference model in Eq. (3.10) is considered. Let ${\text{ V }aR}^{\varvec{\theta }_h}_{1-p} [X_{n+1} | \mathcal{F}^X_n ]$ and ${\text{ E }S}^{\varvec{\theta }_h}_{1-p} [X_{n+1} | \mathcal{F}^X_n ]$ be the VaR and ES of the short position of the Bitcoin in the $(n+1)^{st}$-period given $\mathcal{F}^X_n$ with confidence level $1 - p$ evaluated under the probability measure $\mathbb {P}^{\varvec{\theta }_h}$, respectively. Using the notation in Online Appendix E and Eq. (B.7) in Online Appendix B, ${\text{ V }aR}^{\varvec{\theta }_h}_{1-p} [X_{n+1} | \mathcal{F}^X_n ]$ and ${\text{ E }S}^{\varvec{\theta }_h}_{1-p} [X_{n+1} | \mathcal{F}^X_n ]$ have the following analytical expressions:

5.7 $$\begin{aligned} {\text{ V }aR}^{\varvec{\theta }_h}_{1-p} [X_{n+1} | \mathcal{F}^X_n ] = {\hat{f}} + \mu {\hat{h}} (\mu _h) + \sigma {\hat{h}} (\mu _h) z_{1 - p}, \end{aligned}$$

and

5.8 $$\begin{aligned} {\text{ E }S}^{\varvec{\theta }_h}_{1-p} [X_{n+1} | \mathcal{F}^X_n ] = {\hat{f}} + \mu {\hat{h}} (\mu _h) + \sigma {\hat{h}} (\mu _h) \frac{ \phi (z_{1 - p})}{p}, \end{aligned}$$

where ${\hat{h}} (\mu _h) := {\hat{h}} (X_{n}, X_{n-1}, \ldots , X_{n+1 - q}, {\hat{V}}_{n+1} (\mu _h) )$ as defined in Online Appendix E; ${\hat{V}}_{n+1} (\mu _h)$ is given by Eq. (E.12) in Online Appendix E.

When the reference model is the first-order SV model in Eq. (3.12), ${\text{ V }aR}^{\varvec{\theta }_h}_{1-p} [X_{n+1} | \mathcal{F}^X_n ]$ in Eq. (5.7) and ${\text{ E }S}^{\varvec{\theta }_h}_{1-p} [X_{n+1} | \mathcal{F}^X_n ]$ Eq. (5.8) become:

5.9 $$\begin{aligned} {\text{ V }aR}^{\varvec{\theta }_h}_{1-p} [X_{n+1} | \mathcal{F}^X_n ] = \mu \exp ({\hat{V}}_{n+1} (\mu _h) / 2) + \sigma \exp ({\hat{V}}_{n+1} (\mu _h) / 2) z_{1 - p}, \end{aligned}$$

and

5.10 $$\begin{aligned} {\text{ E }S}^{\varvec{\theta }_h}_{1-p} [X_{n+1} | \mathcal{F}^X_n ] = \mu \exp ({\hat{V}}_{n+1} (\mu _h) / 2) + \sigma \exp ({\hat{V}}_{n+1} (\mu _h) / 2) \frac{ \phi (z_{1 - p})}{p},\nonumber \\ \end{aligned}$$

where ${\hat{V}}_{n+1} (\mu _h)$ is given by Eq. (E.12) in Online Appendix E.

The upper-point estimates, the lower-point estimates and the interval estimates for the VaR and ES under the reference model in Eq. (3.10) are then evaluated in the same fashion as those under the reference model in Eq. (3.1) as described above, with $\varvec{\theta }$ replaced by $\varvec{\theta }_h$, $\varvec{\Theta }_{2 \gamma _1, 2 \gamma _2} (\mathbf{e} (n))$ replaced by $\varvec{\Theta }_{2 \gamma _1, 2 \gamma _2, 2 \gamma _{h1}, 2 \gamma _{h2}} (\mathbf{e} (n), \varvec{\nu }(n))$ and $\varvec{\Theta }_{\gamma _1, \gamma _2} (\mathbf{e} (n))$ replaced by $\varvec{\Theta }_{\gamma _1, \gamma _2, \gamma _{h1}, \gamma _{h2}} (\mathbf{e} (n), \varvec{\nu }(n))$.

The Bayesian risk-neutral estimate for the VaR is given by:

5.11 $$\begin{aligned} {\text{ B }RNVaR}^h_{1-p} [X_{n+1} | \mathcal{F}^X_n ] = {\hat{f}} + \mu _n {\hat{h}} (\mu _{hn}) + \sigma _n {\hat{h}} (\mu _{hn}) z_{1 - p}, \end{aligned}$$

where $\mu _n$, $\mu _{hn}$ and $\sigma _n$ are given by Eq. (4.6), Eq. (C.8) in Online Appendix C and Eq. (4.7), respectively. Under the first-order SV model in Eq. (3.12), the Bayesian risk-neutral estimate in Eq. (5.11) becomes:

5.12 $$\begin{aligned} {\text{ B }RNVaR}^h_{1-p} [X_{n+1} | \mathcal{F}^X_n ] = \mu _n \exp ({\hat{V}}_{n+1} (\mu _{hn}) / 2) \!+\ \sigma _n \exp ({\hat{V}}_{n+1} (\mu _{hn}) / 2) z_{1 - p}.\nonumber \\ \end{aligned}$$

The Bayesian risk-neutral estimates for the ES are evaluated similarly.

Note that the estimates for the VaR and ES described as above represent losses in percentage. As in Tsay (2013), Page 337, the estimates of the VaR and ES in dollar amounts can be approximated by first multiplying the estimates for the VaR and ES described as above with the current dollar amount of one unit of the Bitcoin and then dividing the multiplication by 100. This method will be used to compute the estimates for the VaR and ES of the short position of the Bitcoin in dollar amounts in Sect. 6.

Though the focus of the current paper is parametric nonlinear time series modelling, it may be noted that the proposed two-stage approach may be extended to nonparametric time series modelling. In Online Appendix F, some preliminary discussions on some potential extensions along this line are provided at an intuitive level.

## Applications to forecasting and risk evaluation of Bitcoin

In this section, applications of the proposed two-stage approach for estimation and prediction to forecasting and risk evaluation of Bitcoin are provided using real Bitcoin data including some periods of Covid 19. Three major parametric nonlinear time series models, namely the SETAR model, the GARCH model and the SV model, are used as the reference models in the first stage and are estimated from the Bitcoin data using the methods described in Sect. 5.1. Then the one-step-ahead point and interval forecasts for the Bitcoin return based on the Bayesian nonlinear expectations are computed using the formulas in Online Appendix E. Lastly, one-step-ahead point and interval predictions of VaR and ES based on the Bayesian credible intervals are computed using the formulas in Sect. 5.3. When nonlinear time series models are considered, one may use numerical simulations for computing multiple-step-ahead forecasts for the return as well as multiple-step-ahead predictions of VaR and ES. The formulas presented in Online Appendix E and Sect. 5.3 provide a convenient way to numerically compute the one-step-ahead forecasts and predictions. To provide an illustration of the intuition and basic idea of the proposed two-stage approach, one-step-head forecasts and predictions are considered here for comparing the three models. As noted in Sect. 5.3, risk metrics such as VaR are often computed for each period, say one day, in practice. To study the impacts of the prior parameters (hyperparameters) for the “uncertain” parameters for misspecifications in the drift and volatility of the reference models on forecasting and risk evaluation of Bitcoin, three sets of prior parameters are used. The computations were done using the statistical package R.

Bitcoin is a digital currency, or cryptocurrency. Cryptocurrencies, together with a related technology called Blockchain, seem to be thought of as important innovations in the FinTech world. The term “cryptocurrency” appears to be related to an important branch in mathematics for coding, namely cryptography, which may have been used in mining Bitcoins. Recently, there have been growing in trading activities of Bitcoins around the world. The growth has accelerated in some periods of Covid 19. Nevertheless, it appears that trading Bitcoins may be risky. Empirical studies reveal that the volatility level of Bitcoins is high. See, for example, Baur and Dimpfl (2021) and the relevant literature therein. Consequently, risk measurement and management for trading Bitcoins could be practically relevant issues. Some recent studies employed GARCH-type models to describe the daily volatility of a Bitcoin series. See, for example, Bouoiyour and Selmi (2016), Dyhrberg (2016), Bouri et al. (2017), Katsiampa (2017) and Baur and Dimpfl (2021). The uses of the GARCH-type models and some of their variants to study risk metrics, such as VaR and ES, for Bitcoins or other cryptocurrencies were considered in the literature. See, for example, Chan et al. (2017), Chu et al. (2017), Osterrieder and Lorenz (2017), Stavroyiannis (2017), Colucci (2018), Aslanidis et al. (2019), Caporale and Zekokh (2019), Trucíos (2019) and Siu (2021). Forecasting and risk evaluation of Bitcoin using Bayesian econometrics were also considered in the literature. See, for example, Hotz-Behofsits et al. (2018), Catania et al. (2019) and Philip et al. (2020). In a recent paper by Siu and Elliott (2021), the pricing of Bitcoin options under a SETAR-GARCH model was considered.

**Fig. 1:**
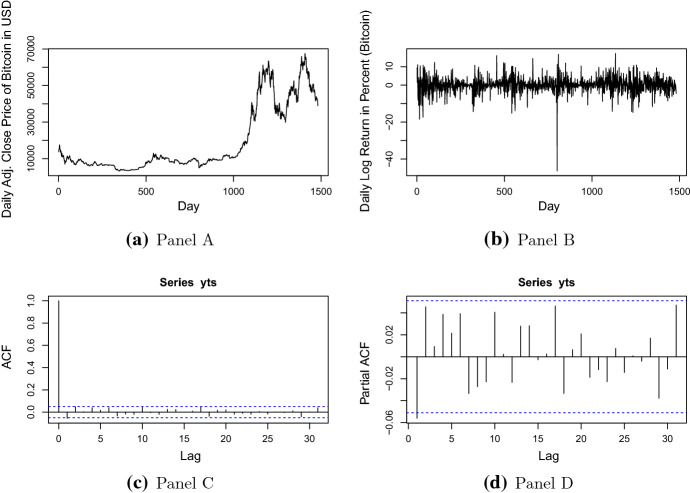
Panel A plots the time series plot of the daily adj. close prices of Bitcoin against US dollars. Panel B depicts the time series plot of the daily percentage logarithmic returns of Bitcoin. Panels C and D plot the sample ACF and sample PACF of the daily percentage logarithmic returns of Bitcoin, respectively

Daily adjusted close prices of Bitcoin in USD from 1 January 2018 to 21 January 2022 with 1,482 observations are used in the empirical study of this paper. The Bitcoin dataset covers some periods of Covid 19. The data were downloaded from Yahoo Finance. To visualize some patterns of the Bitcoin data, we can look at Fig. 1, which gives the time series plots for the daily adjusted close prices, the daily logarithmic returns in percent, the sample ACF and the sample PACF.

From Fig. 1 (Panel A), it is clear that there were sharp upward trends in the daily adjusted close prices in some periods of Covid 19, (i.e. some periods after the 1000th observation. Figure 1 (Panel B) shows that the volatility of the daily logarithmic returns appears to change over time. From Fig. 1 (Panels C and D), the daily logarithmic returns look stationary.

The summary statistics of the daily percentage logarithmic returns from the Bitcoin, which were computed using “basicStats” in the R package “fBasics” (Wuertz et al. 2020), are displayed in Table 1.

From Table 1, it may be seen that the Bitcoin returns data are negatively skewed, (i.e. skewness $-1.141107$) and heavy-tailed, (i.e. kurtosis 14.086406). The range of fluctuations in the returns data are large, (i.e. minimum $-46.473018$ and maximum 17.182056).

For illustration, a SETAR(2,1,1) model with $d = 1$, a GARCH(1,1) model and a first-order SV model are used as three reference models for the daily percentage logarithmic returns of the Bitcoin. The SETAR(2,1,1) model with $d = 1$ is given by:

6.1 $$\begin{aligned} X_t = ( \alpha ^{(1)}_0 + \alpha ^{(1)}_1 X_{t-1} + \epsilon _t ) I_{\{ X_{t-1} \le r \}} + ( \alpha ^{(2)}_0 + \alpha ^{(2)}_1 X_{t-1} + \epsilon _t ) I_{\{ X_{t-1} > r \}}, \end{aligned}$$

where $I_E$ is the indicator function of an event *E*; *r* is the threshold parameter. The SETAR(2,1,1) model in Eq. (6.1) is the reference model in Eq. (3.1) with the conditional mean function $f = ( \alpha ^{(1)}_0 + \alpha ^{(1)}_1 X_{t-1} + \epsilon _t ) I_{\{ X_{t-1} \le r \}} + ( \alpha ^{(2)}_0 + \alpha ^{(2)}_1 X_{t-1} + \epsilon _t ) I_{\{ X_{t-1} > r \}}$ and the volatility function $g = 1$.

The GARCH(1,1) model is given by:

6.2 $$\begin{aligned} X_t= & {} \sqrt{h_t} \epsilon _t, \nonumber \\ h_t= & {} \gamma _0 + \gamma _1 X^2_{t-1} + \delta h_{t-1}. \end{aligned}$$

The GARCH(1,1) model in Eq. (6.2) is the reference model in Eq. (3.1) with the conditional mean function $f = 0$ and the conditional volatility function $g = \sqrt{h_t}$. The first-order SV model is given by Eq. (3.12), which is included the reference model in Eq. (3.10), as described in Sect. 3.2.

The estimation results for the SETAR(2,1,1) model, the GARCH(1,1) model and the first-order SV model are presented in Tables 2, 3 and 4, respectively. The estimation of the SETAR(2,1,1) model was done by the function “tar” in the R package “TSA” (Chan and Ripley 2020). The testing of the SETAR(2,1,1) model based on the Threshold nonlinearity test in Tsay (1989) was done using the function “thr.test” in the R package “NTS” (Tsay et al. 2020). The estimation and testing of the GARCH(1,1) model were done using the function “garch” in the R package “tseries” (Trapletti et al. 2021), where the testing of the GARCH(1,1) model is based on the Box–Ljung test for the squared residuals. The R codes were written to estimate the first-order SV model using the QMLE with the Kalman filter (see Appendix B of Jacquier et al. (2002)). The R function “nlminb” was used in the optimization of the QMLE with the initial values of the parameters: $\alpha _v = 0.05$, $\delta _v = 0.9$ and $\sigma _v = 0.2$.

From Table 2, the result of the Threshold nonlinearity test provides evidence to support the SETAR(2,1,1) model with $d = 1$, (i.e. *p*-value $= 0.01489898$), at $5\%$ significance level. From Table 3, the result of the Box–Ljung test for the squared residuals indicates the presence of the ARCH effect, (i.e. *p*-value $= 0.6965$). Comparing the log-likelihood values of the three models, the first-order SV model provides the best fit to the Bitcoin data, which gives the largest log-likelihood value of $-3443.831$.

Now three sets of prior parameters (hyperparameters) are considered. The first set of prior parameters (Set 1) is: $\mu _0 = 0.05$, $t_0 = 100$, $\mu _{h0} = 0.05$, $t_{h0} = 100$, $\alpha = 2$, $\beta = 1$, $\alpha _h = 2$ and $\beta _h = 1$. The second set of prior parameters (Set 2) is: $\mu _0 = 0$, $t_0 = 10$, $\mu _{h0} = 0.05$, $t_{h0} = 100$, $\alpha = 1$, $\beta = 1$, $\alpha _h = 2$ and $\beta _h = 1$. Under Set 2, the prior means for $\mu$ and $\sigma$ are 0 and 1, respectively. This indicates that according to the prior estimates given by the prior means for $\mu$ and $\sigma$, there are no misspecifications in the conditional mean and volatility of a reference model given by either the SETAR(2,1,1) model in Eq. (6.1) or the GARCH(1,1) model in Eq.(6.2), and there are no misspecifications in the conditional mean and volatility of the observation process in a reference model given by the first-order SV model in Eq. (3.12). According to the prior precisions for $\mu$ and $\sigma$, there is less (higher) confidence on the prior mean for $\mu$ ($\sigma$) under Set 2 than Set 1. The third set of prior parameters (Set 3) is: $\mu _0 = 0.5$, $t_0 = 100$, $\mu _{h0} = 0.5$, $t_{h0} = 100$, $\alpha = 1$, $\beta = 2$, $\alpha _h = 1$ and $\beta _h = 1$. Under Set 3, the prior mean for $\sigma _h$ is 1. This indicates that according to the prior estimate given by the prior mean for $\sigma _h$, there is no misspecification in the volatility of the latent process in a reference model given by the first-order SV model in Eq. (3.12). According to the prior estimates given by the prior means for $\mu$ and $\mu _h$, there are higher levels of misspecifications in the conditional means $\mu$ and $\mu _h$ of the respective reference models under Set 3 than Set 1. According to the prior precisions for $\sigma$ and $\sigma _h$, there is higher confidence on the prior means for $\sigma$ and $\sigma _h$ under Set 3 than Set 1.

Table 5 presents the 95% Bayesian credible intervals (BCIs) and the Bayesian risk-neutral estimates (BRNEs) for the “uncertain” parameters $(\mu , \sigma )$ of the SETAR(2,1,1) model in Eq. (6.1) and the GARCH(1,1) model in Eq.(6.2) under the prior parameters in Set 1. Table 6 gives the $95\%$ BCIs and the BRNEs for the “uncertain” parameters $(\mu , \sigma , \mu _h, \sigma _h)$ of the SV model in Eq. (3.12) under the prior parameters in Set 1. All the BCIs and BRNEs are expressed as percentage.

From Table 5, according to the BRNEs, the misspecification in the volatility $\sigma$ under the GARCH(1,1) model is less severe than the SETAR(2,1,1) model. Also, the misspecification in the conditional mean $\mu$ under the SETAR(2,1,1) model is less severe than the GARCH(1,1) model. These results are consistent with intuition because the SETAR(2,1,1) model incorporates nonlinearity such as regime switching in the conditional mean while the GARCH(1,1) model captures changing volatility, say the conditional heteroscedasticity. According to the BCIs, the interval estimates for the misspecifications in the volatilities of the GARCH(1,1) model and the SETAR(2,1,1) model are precise. However, the interval estimate for the misspecifications in the conditional means of the SETAR(2, 1, 1) model and the GARCH(1,1) model are not precise. In view of the Bitcoin data, there were sharp upward trends in the daily adjusted close prices in some periods of Covid 19 as depicted in the time series plot in Fig. 1 (Panel A). Consequently, modelling and predicting the conditional mean of the Bitcoin return data become challenging. It is difficult to give a precise estimation for the misspecification in the conditional mean, which is reflected in the wider interval estimates for the misspecifications in the conditional means by the BCIs. This appears to be in line with Merton (1980), where it was noted that the drift of financial returns was uneasy to be precisely estimated. From Table 6, according to the BRNEs, the misspecifications in the conditional mean and volatility of the observation process under the first-order SV model are slightly more severe than the GARCH(1,1) model. However, the misspecifications on the conditional mean and volatility of the observation process are more severe than the latent process under the SV model. This is consistent with the intuition because it may be more difficult to specify a latent process, which is unobservable, correctly than an observable process. Furthermore, according to the BCIs, the interval estimates for the misspecifications in the conditional means of both the observation and latent processes are less precise than the interval estimates for the misspecifications in the volatility of the two processes under the SV model. This is consistent with the respective results for the SETAR(2,1,1) model and the GARCH(1,1) model.

Table 7 gives the one-step-ahead forecasts, say an upper-point forecast (UPF), a lower-point forecast (LPF), an interval forecast (IF), a Bayesian risk-neutral forecast (BRNF) and a classical forecast (CF) based on a reference model only, of the daily percentage logarithmic returns from the SETAR(2,1,1) model, the GARCH(1,1) model and the first-order SV model estimated from the Bitcoin data under the prior parameters in Set 1. All the forecasts using the Bayesian nonlinear expectations, namely UPF, LPF and IF, were evaluated at the 95% probability level for the “uncertain” parameters $(\mu , \sigma , \mu _h, \sigma _h)$. The one-step-head forecasts were expressed as percentage and were computed based on the Bitcoin data in the whole sampling period from 1 January 2018 to 21 January 2022 with 1,482 observations.

From Table 7, the one-step-ahead forecasts from the GARCH(1,1) model and the SV model are generally lower than those from the SETAR(2,1,1) model. This is consistent with the assumption that the conditional means of the reference models given by the GARCH(1,1) model and the SV model are zero. From the LPF, UPF and IF from the GARCH(1,1) model and the SV model, it may be seen that the drift and volatility misspecifications have a more pronounced impact on the one-step-ahead forecasts from the SV model than those from the GARCH(1,1) model. This is also evidenced by comparing the BRNF and CF from each of the GARCH(1,1) model and the SV model. To explain this, it may be noted that the drift and volatility misspecifications are incorporated in both the observation and latent processes under the SV model, while the drift and volatility misspecifications are captured in the observation process only under the GARCH(1,1) model. Lastly, the difference between the BRNE and the CF under the SETAR(2,1,1) model is small, while the difference between the UPF and the LPF under the SETAR(2,1,1) model is large. This is consistent with the results in Table 5 that the BRNE for $\mu$ is small and the 95% BCI for $\mu$ is wide under the SETAR(2,1,1) model.

Table 8 gives the one-step-ahead point and interval predictions for the two risk metrics, namely VaR and ES, for the short position of one unit of the Bitcoin from the SETAR(2,1,1) model, the GARCH(1,1) model and the SV model estimated from the Bitcoin data under the prior parameters in Set 1. Specifically, an upper-point VaR prediction (UVaR), an upper-point ES prediction (UES), a lower-point VaR prediction (LVaR), a lower-point ES prediction (LES), an interval VaR prediction (IVaR), an interval ES prediction (IES), a Bayesian risk-neutral VaR prediction (BRNVaR), a Bayesian risk-neutral ES prediction (BRNES), a classical VaR prediction (VaR) and a classical ES prediction (ES) based on the respective reference model only are presented. All the estimates of the two risk metrics based on the Bayesian nonlinear expectations, namely the UVaR, UES, LVaR, LES, IVaR and IES, were evaluated at the 95% probability level for the “uncertain” parameters $$(\mu , \sigma , \mu _h, \sigma _h)$$. The confidence levels for the VaR and ES are both equal to $$95\%$$, (i.e. $$p = 0.05$$). All the estimates of the two risk metrics are expressed in USD. The one-step-ahead VaR and ES predictions were computed based on the Bitcoin data in the whole sampling period from 1 January 2018 to 21 January 2022 with 1,482 observations. The adjusted close price of one unit of Bitcoin in USD on 21 January 2022 was $38889.68. To numerically compute the UVaR, UES, LVaR, LES, IVaR and IES, a set of 11 grid points is used to equally partition the 95% BCI for each of the “uncertain” parameters $$(\mu , \sigma , \mu _h, \sigma _h)$$.

From Table 8, with the drift and volatility misspecifications incorporated, the SETAR(2,1,1) model gives the most conservative one-step-ahead VaR and ES predictions among the three models, while the GARCH(1,1) model gives the least conservative one-step-ahead VaR and ES predictions. However, without incorporating the drift and volatility misspecifications, the SV model gives the most conservative one-step-ahead VaR and ES predictions among the three models, while the SETAR(2,1,1) model gives the least conservative one-step-ahead VaR and ES predictions. Unlike the one-step-ahead forecasts for the Bitcoin return, where the conditional mean of the observation process is the key factor, the volatility of the observation process is the key factor for the one-step-ahead VaR and ES predictions. From Tables 5-6, the SETAR(2,1,1) model has the most severe misspecifications in the volatility of the observation process among the three model, while the GARCH(1,1) model has the least severe misspecification in the volatility of the observation process. Consequently, the results from Table 8 indicate that the more severe the misspecification in the volatility of the observation process is, the more conservative the one-step-ahead risk prediction is.

The results based on different sets of prior parameters (hyperparameters), say Set 2 and Set 3, are presented in Online Appendix G. It may be seen that the results under the prior parameters in Set 2 and Set 3 are qualitatively similar to those under the prior parameters in Set 1. This indicates that the observations and conclusions from the results under the prior parameters in Set 1 may be quite robust.

## Conclusions

A two-stage approach was proposed to parametric time series modelling with the drift and volatility misspecifications or uncertainties. A reference model is specified and estimated in the first stage. At the second stage, the Bayesian nonlinear expectations were used to incorporate a family of alternative models for prediction, where the family alternative models were introduced using discrete-time Girsanov’s transforms. The idea is to make use of the information that is left over in the residuals of the estimated reference model to construct Bayesian credible intervals for the “uncertain” parameters describing the drift and volatility misspecifications. Using conjugate priors, closed-form Bayesian credible intervals are obtained, which provide a convenient way to construct the Bayesian nonlinear expectations used for prediction so that probability levels are assigned to the nonlinear expectations. The closed-form Bayesian credible intervals combine both a modeller’s prior/subjective belief (or expert opinion) and the objective data in estimating the drift and volatility misspecifications. The prior/subjective belief may be useful to make adjustments for prediction in case the future developments may not follow the current and past data. To introduce the two-stage approach, two general classes of parametric nonlinear time series models, namely a nonlinear autoregressive model with conditional heteroscedasticity and a product process, are considered. The former includes the SETAR model and the GARCH model, while the latter includes the SV model. The link between the Bayesian shrinkage and regularization techniques for estimation and variable selection and the Bayesian nonlinear expectations was discussed in Online Appendix D. Some potential generalizations based on both classical and Bayesian nonparametric approaches were outlined in Online Appendix F. Applications of the proposed two-stage approach to forecasting and risk evaluation of Bitcoin were illustrated empirically using real Bitcoin data covering some periods of Covid 19 and the three models, namely the SETAR model, the GARCH model and the SV model. The results highlight the importance of incorporating misspecifications in the conditional mean and volatility for making one-step-ahead forecasts for the Bitcoin return and one-step-ahead VaR and ES predictions for the short position of the Bitcoin, respectively.

### Supplementary Information

Below is the link to the electronic supplementary material.

Supplementary file 1 (pdf 333 KB)

## A Appendix

See Tables 1, 2, 3, 4, 5, 6, 7 and 8.

**Table 1:** Summary statistics of the daily percentage log returns

nobs	Mean	Stdev	Skewness	Kurtosis
1481	0.070659	4.005866	$$-1.141107 $$	14.086406
Minimum	1 Quartile	Median	3 Quartile	Maximum
$$-46.473018 $$	$$-1.572926$$	0.136811	1.836281	17.182056

**Table 2:** Estimation and testing results of the SETAR(2,1,1) model with ($d = 1$)

$\alpha ^{(1)}_0$	$\alpha ^{(1)}_1$	$\alpha ^{(2)}_0$	$\alpha ^{(2)}_1$
$$ -0.2141628$$	$$ -0.1214365 $$	0.01773623	0
*r*	log-like	*F*-ratio	*p*-value
0.02149885	$$-4141.243 $$	4.218792	0.01489898

**Table 3:** Estimation and testing results of the GARCH(1,1) model

$\gamma _0$	$\gamma _1$	$\delta$	log-like	*X*-squared	*p*-value
0.97348	0.08517	0.85911	$-4087.579$	0.15217	0.6965

**Table 4:** Estimation results of the SV model

$\alpha _v$	$\delta _v$	$\sigma _v$	log-like
0.05157367	0.97363784	0.23617299	$-3443.831$

**Table 5:** 95% BCIs and BRNEs for $\mu$ and $\sigma$ (Set 1 priors)

	95% BCI (SETAR)	BRNE (SETAR)	95% BCI (GARCH)	BRNE (GARCH)
$\mu$	($-0.1617407$, 0.1680658)	0.003162555	($-0.02291122$, 0.05989249)	0.01849063
$\sigma$	(3.867361, 4.108072)	3.983795	(0.9709686, 1.031403)	1.000201

**Table 6:** 95% BCIs and BRNEs for $\mu$, $\sigma$, $\mu _h$ and $\sigma _h$ (Set 1 priors)

	95% BCI (SV)	BRNE (SV)
$\mu$	($-0.04263042$, 0.08687772)	0.02212365
$\sigma$	(1.518632, 1.613154)	1.564353
$\mu _h$	(0.001556987, 0.004768124)	0.003162555
$\sigma _h$	(0.03765428, 0.03999794)	0.03878793

**Table 7:** One-step-ahead forecasts (Set 1 priors)

	SETAR	GARCH	SV
LPF	0.1707797	$$ -0.03527248 $$	$$ -0.09918374$$
UPF	0.5005863	0.1007441	0.2022059
IF	(0.139151, 0.532215)	($-0.04831657$, 0.1137882)	($-0.1280752$, 0.2311215)
BRNF	0.335683	0.03273581	0.05148254
CF	0.3325205	0	0

**Table 8:** Estimates of VaR and ES (Set 1 priors)

	SETAR	GARCH	SV
UVaR	2822.521	1123.269	2480.36
UES	3490.099	1398.671	3090.495
LVaR	2540.282	1006.871	2221.565
LES	3168.744	1266.142	2795.732
IVaR	(2514.025, 2850.405)	(996.0422, 1134.768)	(2197.497, 2505.936)
IES	(3138.941, 3521.943)	(1253.851, 1411.803)	(2768.403, 3119.712)
BRNVaR	2678.893	1064.036	2348.646
BRNES	3326.276	1331.109	2940.211
VaR	768.9944	1049.765	1487.999
ES	931.4985	1316.447	1866.011

## B Appendix

See Fig. 1.

## Footnotes

1 The term “a baseline model” was used in Hansen and Sargent (2021).

2 In general one may consider a multiple time series process.
