# Supplementary material for: Bayesian nonlinear expectation for time series modelling and its application to Bitcoin
Source: Empir Econ. 2022 May 25;64(1):505–37. doi: 10.1007/s00181-022-02255-z (PMC9130704; doi:10.1007/s00181-022-02255-z)
Supplement: Supplementary file 1 — (pdf 333 KB) [file 181_2022_2255_MOESM1_ESM.pdf]

Bayesian Nonlinear Expectation for Time Series Modelling and Its Application to Bitcoin

Tak Kuen Siu *

April 21, 2022

Journal: Empirical Economics

Supplementary Material: Online Appendices A-G

*Department of Actuarial Studies and Business Analytics, Macquarie Business School, Macquarie University, Sydney, NSW 2109, Australia; Email: Ken.Siu@mq.edu.au; ktksiu2005@gmail.com; Tel.: (+61-2) 9850 8589.

A g-Expectations, G-Expectations and Model Uncertainty

Some technical details on two types of nonlinear expectations, namely the g-expectation and the G-expectation, and their links with model uncertainty or ambiguity are provided. Firstly, the notion of (conditional) nonlinear expectations is defined along the lines of Peng (2004, 2006) and Rosazza Gianin (2006). Let $\mathcal{R}$ be a space of random variables defined on a complete probability space $(\Omega, \mathcal{F}, \mathbb{P})$, where $\mathcal{F}$ is a σ-field on the sample space Ω and $\mathbb{P}$ is a reference probability measure. Let $\mathcal{H}$ be a sub-σ-field of $\mathcal{F}$, (i.e., $\mathcal{H} \subset \mathcal{F}$). Write $\mathcal{R}_{\mathcal{H}}$ for the space of random variables which are measurable with respect to $\mathcal{H}$. Then a (conditional) nonlinear expectation given $\mathcal{H}$ is defined as follows:

Definition A.1. *A conditional nonlinear expectation $\mathcal{E}[\cdot|\mathcal{H}]$ given $\mathcal{H}$ is a functional $\mathcal{R} \to \mathcal{R}_{\mathcal{H}}$ which satisfies the following properties:*

(a) **Measurability:** *$\mathcal{E}[X|\mathcal{H}] = X$ if $X \in \mathcal{R}_{\mathcal{H}}$, (i.e., the random variable X is $\mathcal{H}$-measurable).*

(b) **Monotonicity:** *If $X, Y \in \mathcal{R}$ and $X := X(\omega) \geqslant Y := Y(\omega)$, for all $\omega \in \Omega$, then $\mathcal{E}[X|\mathcal{H}] \geqslant \mathcal{E}[Y|\mathcal{H}]$.*

(c) **Translation Invariance:** *If $X \in \mathcal{R}$ and $Y \in \mathcal{R}_{\mathcal{H}}$, then $\mathcal{E}[X+Y|\mathcal{H}] = \mathcal{E}[X|\mathcal{H}]+Y$.*

(d) **Positive Homogeneity:** *If $X \in \mathcal{R}$ and $\lambda \in \mathcal{R}_{\mathcal{H}}$ such that $\lambda := \lambda(\omega) \geqslant 0$, for all $\omega \in \Omega$, then $\mathcal{E}[\lambda X|\mathcal{H}] = \lambda \mathcal{E}[X|\mathcal{H}]$.*

(e) **Sub-additivity:** *If $X, Y \in \mathcal{R}$, then $\mathcal{E}[X + Y|\mathcal{H}] \leqslant \mathcal{E}[X|\mathcal{H}] + \mathcal{E}[Y|\mathcal{H}]$.*

The notion of nonlinear expectations is closely linked with the concept of coherent risk measures in Artzner et al. (1999). Specifically, a (conditional) coherent risk measure also satisfies Properties (c)-(e) in Definition A.1. A (conditional) nonlinear expectation satisfying the sub-additivity property, (i.e., Property (e) in Definition A.1), is called a (conditional) sublinear expectation. A (conditional) expectation satisfying the super-additivity property, (i.e., Property (e) in Definition A.1 with "$\leqslant$" replaced with "$\geqslant$"), is called a (conditional) superlinear expectation.

In a continuous-time modelling framework, a conditional g-expectation is defined by a solution of a backward stochastic differential equation. To illustrate this, the expositions in Chen and Epstein (2002) are followed. Let $\{W_t | t \in [0, T]\}$ be a (real)-valued standard Brownian motion defined on $(\Omega, \mathcal{F}, \mathbb{P})$, where $[0, T]$ is a finite horizon, (i.e., $T < \infty$). Write $\mathbb{F}^W$ for the filtration $\{\mathcal{F}^W_t | t \in [0, T]\}$, where $\mathcal{F}^W_t$ is the $\mathbb{P}$-completed σ-field generated by the $\{W_u | u \in [0, t]\}$. Let $L^2(\Omega, \mathcal{F}^W_T, \mathbb{P})$ be the space of $\mathcal{F}^W_T$-measurable, square-integrable variables with respect to $\mathbb{P}$. Let $\{\theta_t | t \in [0, T]\}$ be an $\mathbb{F}^W$-adapted, real-valued

process defined on $(\Omega, \mathcal{F}, \mathbb{P})$. Write Θ for the space of such processes $\{\theta_t | t \in [0, T]\}$. Consider the following backward stochastic differential equation:

$$dY_t = \big(\max_{\theta \in \Theta} \theta_t \cdot \sigma_t\big) dt + \sigma_t dW_t, \quad Y_T = \xi. \tag{A.1}$$

Note that from Eq. (A.1), Y_t depends on Θ, (i.e., $Y_t = Y_t^{\Theta}$). However, to simplify the notation, we write Y_t for Y_t^{Θ}.

For each $t \in [0, T]$, let $L^2(\Omega, \mathcal{F}_t^W, \mathbb{P})$ be the space of $\mathcal{F}_t^W$-measurable, square-integrable variables with respect to $\mathbb{P}$. Define, for each $t \in [0, T]$, a nonlinear functional $\mathcal{E}_{\Theta}[\cdot | \mathcal{F}_t^W] : L^2(\Omega, \mathcal{F}_T^W, \mathbb{P}) \to L^2(\Omega, \mathcal{F}_t^W, \mathbb{P})$ with respect to Θ by $\mathcal{E}_{\Theta}[\xi | \mathcal{F}_t^W] := Y_t$. Then as noted in Chen and Epstein (2002),

$$\mathcal{E}_{\Theta}[\xi | \mathcal{F}_t^W] := Y_t = \min_{\theta \in \Theta} \mathrm{E}^{\theta}[\xi | \mathcal{F}_t^W], \tag{A.2}$$

where $\mathrm{E}^{\theta}[\cdot | \mathcal{F}_t^W]$ is the conditional expectation under a probability measure $\mathbb{P}^{\theta}$ given $\mathcal{F}_t^W$, for each $\theta \in \Theta$. It may not be difficult to see that $\mathcal{E}_{\Theta}[\xi | \mathcal{F}_t^W]$ in Eq. (A.2) satisfies the properties in Definition A.1 with the sub-additivity replaced with super-additivity, and hence, it is a conditional nonlinear (or superlinear) expectation. Indeed, $\mathcal{E}_{\Theta}[\xi | \mathcal{F}_t^W]$ in Eq. (A.2) is a conditional g-expectation, which describes the drift uncertainty characterized by $\theta \in \Theta$ as can be seen from Eq. (A.1). For each $\theta \in \Theta$, the probability measure $\mathbb{P}^{\theta}$ can be defined by the continuous-time standard Girsanov's transform for Brownian motions:

$$\frac{d\mathbb{P}^{\theta}}{d\mathbb{P}}\bigg|_{\mathcal{F}_T^W} := \exp\left(-\int_0^T \theta_t dW_t - \frac{1}{2} \int_0^T \theta_t^2 dt \right), \tag{A.3}$$

where the process $\{\theta_t | t \in [0, T]\}$, which is called a density generator, satisfies a technical condition, (i.e., the Novikov's condition). Then a family $\{\mathbb{P}^{\theta} | \theta \in \Theta\}$ of probability measures absolutely continuous with respect to the reference probability measure $\mathbb{P}$ can be defined. This family $\{\mathbb{P}^{\theta} | \theta \in \Theta\}$ defines a family of alternative models describing the model uncertainty or ambiguity about the drift of a process. As noted by Chen and Epstein (2002), the difference $\mathrm{E}[\xi | \mathcal{F}_t^W] - \mathrm{E}^{\theta}[\cdot | \mathcal{F}_t^W]$ may be interpreted as a premium attributed to model uncertainty or ambiguity, where $\mathrm{E}[| \mathcal{F}_t^W]$ is the conditional expectation under the reference probability measure $\mathbb{P}$ given $\mathcal{F}_t^W$.

To describe a conditional G-expectation in continuous time, the expositions in Fouque and Ren (2014) are followed, where option valuation under volatility uncertainty was considered. Suppose that under a risk-neutral probability measure $\mathbb{Q}$, the price process $\{S_t | t \in [0, T]\}$ of a risky asset follows a stochastic differential equation:

$$dS_t = rS_t dt + \sigma_t dW_t, \tag{A.4}$$

where r is the constant continuously compounded risk-free interest rate; $\{W_t | t \in [0, T]\}$ is the standard Brownian motion as defined before; the "uncertain" volatility process

$\{\sigma_t | t \in [0,T]\}$ is $\mathbb{F}^W$-progressive measurable with state space given by a bounded interval $[\sigma_L, \sigma_U]$, where $\sigma_L, \sigma_U > 0$ and $\sigma_L < \sigma_U$. Write Θ_σ for the space of all such processes $\{\sigma_t | t \in [0,T]\}$. Consider a European option written on the risky asset S with the payoff function $V(S_T)$ at maturity T. Then the conditional "worst-case" scenario price of the European option at time t given $\mathcal{F}_t^W$ is:

$$V_t = \text{ess} - \sup_{\theta_\sigma \in \Theta_\sigma} \mathrm{E}^{\theta_\sigma}[e^{-r(T-t)} V(S_T) | \mathcal{F}_t^W], \tag{A.5}$$

where $\mathrm{E}^{\theta_\sigma}[\cdot | \mathcal{F}_t^W]$ is the conditional expectation under a probability measure $\mathbb{P}^{\theta_\sigma}$ given $\mathcal{F}_t^W$, for each $\theta_\sigma \in \Theta_\sigma$; ess $-$ sup is the essential supremum. The conditional "worst-case" scenario price in Eq. (A.5) is interpreted as the super-replication price of the European option from the option's seller perspective. Define, for each $t \in [0,T]$, a nonlinear functional $\mathcal{E}_{\Theta_\sigma}(\cdot | \mathcal{F}_t^W)$ corresponding to the conditional "worst-case" scenario price in Eq. (A.5) as follows:

$$\mathcal{E}_{\Theta_\sigma}(\xi | \mathcal{F}_t^W) := \text{ess} - \sup_{\theta_\sigma \in \Theta_\sigma} \mathrm{E}^{\theta_\sigma}[\xi | \mathcal{F}_t^W], \tag{A.6}$$

where ξ is an $\mathcal{F}_T^W$-measurable random variable. Then the nonlinear functional in Eq. (A.6) satisfies the properties in Definition A.1. Consequently, it is a conditional nonlinear (or sublinear) expectation. Indeed, the nonlinear functional in Eq. (A.6) is a conditional G-expectation, which describes the volatility uncertainty characterized by $\theta_\sigma \in \Theta_\sigma$. Similarly, the conditional "best-case" scenario price of the European option at time t given $\mathcal{F}_t^W$ is:

$$V_t = \text{ess} - \inf_{\theta_\sigma \in \Theta_\sigma} \mathrm{E}^{\theta_\sigma}[e^{-r(T-t)} V(S_T) | \mathcal{F}_t^W], \tag{A.7}$$

where ess $-$ inf is the essential infimum. Define, for each $t \in [0,T]$, a nonlinear functional $\mathcal{E}^I_{\Theta_\sigma}(\cdot | \mathcal{F}_t^W)$ corresponding to the conditional "best-case" scenario price in Eq. (A.7) as follows:

$$\mathcal{E}^I_{\Theta_\sigma}(\xi | \mathcal{F}_t^W) := \text{ess} - \inf_{\theta_\sigma \in \Theta_\sigma} \mathrm{E}^{\theta_\sigma}[\xi | \mathcal{F}_t^W]. \tag{A.8}$$

This is a conditional superlinear expectation. The difference $\mathcal{E}_{\Theta_\sigma}(\xi | \mathcal{F}_t^W) - \mathcal{E}^I_{\Theta_\sigma}(\xi | \mathcal{F}_t^W)$ describes the impact of volatility uncertainty (Cont (2006) and Fouque and Ren (2014)). Unlike the conditional g-expectation, the family of probability measures $\{\mathbb{P}^{\theta_\sigma} | \theta_\sigma \in \Theta_\sigma\}$ underlying the conditional G-expectation cannot be defined using the continuous-time standard Girsanov's transform in Eq. (A.3). In fact, for each $\theta_\sigma \in \Theta_\sigma$, the probability measure $\mathbb{P}^{\theta_\sigma}$ is singular to the reference probability measure $\mathbb{P}$.

B A product process

The derivations of a family of alternative models from the reference model in Eq. (3.10) for prediction in the second stage and the respective conditional sublinear and superlinear expectations are provided here. To take into account the presence of the latent process $\{V_t\}_{t\in\mathbb{T}}$, an enlarged information structure is needed. Let $\mathbb{F}^V$ denote the $\mathbb{P}$-augmentation of the natural filtration $\{\mathcal{F}_t^V\}_{t\in\mathbb{T}}$ generated by the latent process $\{V_t\}_{t\in\mathbb{T}}$. That is, for each $t \in \mathbb{T}$, $\mathcal{F}_t^V$ represents the $\mathbb{P}$-completed σ-field generated by the sequence $\{V_1, V_2, \cdots, V_t\}$ of random variables. Write $\mathbb{G}$ for the enlarged filtration $\{\mathcal{G}_t\}_{t\in\mathbb{T}}$ so that for each $t \in \mathbb{T}$, $\mathcal{G}_t = \mathcal{F}_t^X \vee \mathcal{F}_t^V$, (i.e., the minimal σ-field containing both $\mathcal{F}_t^X$ and $\mathcal{F}_t^V$). It may be noted that for each $t \in \mathbb{T}$, $\mathcal{G}_t$ describes the information about the time series process $\{X_t\}_{t\in\mathbb{T}}$ and the latent process $\{V_t\}_{t\in\mathbb{T}}$ up to and including time t.

Similar to Section 3.1, let

$$\boldsymbol{\theta}_h := (\mu, \sigma, \mu_h, \sigma_h)' = (\mu(\boldsymbol{\theta}_h), \sigma(\boldsymbol{\theta}_h), \mu_h(\boldsymbol{\theta}_h), \sigma_h(\boldsymbol{\theta}_h))' \in \Re \times \Re_+ \times \Re \times \Re_+, \tag{B.1}$$

where μ and μ_h may be interpreted as misspecifications in the conditional means (or drifts) in the time series process $\{X_t\}_{t\in\mathbb{T}}$ and the latent process $\{V_t\}_{t\in\mathbb{T}}$, respectively. Likewise, σ and σ_h may be interpreted as misspecifications in the volatility in the time series process $\{X_t\}_{t\in\mathbb{T}}$ and the latent process $\{V_t\}_{t\in\mathbb{T}}$, respectively. Note that μ, σ, μ_h and σ_h are "uncertain" parameters. Again, if $\mu_h = 0$ and $\sigma_h = 1$, there will be no misspecifications in the conditional mean (or drift) and the volatility in the latent process $\{V_t\}_{t\in\mathbb{T}}$, respectively.

Define the product intervals:

$$\boldsymbol{\Theta}_h := [\mu^-, \mu^+] \times [\sigma^-, \sigma^+] \times [\mu_h^-, \mu_h^+] \times [\sigma_h^-, \sigma_h^+], \tag{B.2}$$

for some $\mu^-, \mu^+, \mu_h^-, \mu_h^+ \in \Re$ and $\sigma^-, \sigma^+, \sigma_h^-, \sigma_h^+ \in \Re_+$ with $\mu^- < \mu^+$, $\sigma^- < \sigma^+$, $\mu_h^- < \mu_h^+$ and $\sigma_h^- < \sigma_h^+$. Then, the family $\{(\mu(\boldsymbol{\theta}_h), \sigma(\boldsymbol{\theta}_h), \mu_h(\boldsymbol{\theta}_h), \sigma_h(\boldsymbol{\theta}_h)) | \boldsymbol{\theta}_h \in \boldsymbol{\Theta}_h\}$ may be used to describe the "uncertain" parameters $(\mu, \sigma, \mu_h, \sigma_h)$.

Let $\phi_{\mu_h,\sigma_h}(x)$ denote the pdf of a normal distribution $N(\mu_h, \sigma_h^2)$ with mean μ_h and variance σ_h^2. For each $\boldsymbol{\theta}_h \in \boldsymbol{\Theta}_h$, let $\{\lambda_t(\boldsymbol{\theta}_h)\}_{t\in\mathbb{T}}$ be a $\mathbb{G}$-adapted process on $(\Omega, \mathcal{F}, \mathbb{P})$ defined by:

$$\lambda_t(\boldsymbol{\theta}_h) := \frac{\phi_{\mu,\sigma}(\epsilon_t)}{\phi(\epsilon_t)} \cdot \frac{\phi_{\mu_h,\sigma_h}(\eta_t)}{\phi(\eta_t)} = \frac{\phi(\frac{\epsilon_t - \mu}{\sigma})}{\sigma\phi(\epsilon_t)} \cdot \frac{\phi(\frac{\eta_t - \mu_h}{\sigma_h})}{\sigma_h\phi(\eta_t)}. \tag{B.3}$$

Consider, for each $\boldsymbol{\theta}_h \in \boldsymbol{\Theta}_h$, the following $\mathbb{G}$-adapted process $\{\Lambda_t(\boldsymbol{\theta}_h)\}_{t\in\mathbb{T}}$:

$$\Lambda_t(\boldsymbol{\theta}_h) := \prod_{k=1}^{t} \lambda_k(\boldsymbol{\theta}_h). \tag{B.4}$$

Note that, for each $\boldsymbol{\theta}_h \in \boldsymbol{\Theta}_h$, $\{\Lambda_t(\boldsymbol{\theta}_h)\}_{t\in\mathbb{T}}$ is a $(\mathbb{G}, \mathbb{P})$-martingale. Consequently, for each $\boldsymbol{\theta}_h \in \boldsymbol{\Theta}_h$, a new probability measure $\mathbb{P}^{\boldsymbol{\theta}_h}$ equivalent to $\mathbb{P}$ on $\mathcal{G}_T$ can be defined by:

$$\left.\frac{d\mathbb{P}^{\boldsymbol{\theta}_h}}{d\mathbb{P}}\right|_{\mathcal{G}_T} := \Lambda_T(\boldsymbol{\theta}_h). \tag{B.5}$$

Again, using a discrete-time Girsanov's theorem, for each $\boldsymbol{\theta}_h \in \boldsymbol{\Theta}_h$, under the new probability measure $\mathbb{P}^{\boldsymbol{\theta}_h}$, $\{\epsilon_t(\boldsymbol{\theta}_h)\}_{t\in\mathbb{T}}$ and $\{\eta_t(\boldsymbol{\theta}_h)\}_{t\in\mathbb{T}}$ defined by putting:

$$\epsilon_t(\boldsymbol{\theta}_h) := \frac{\epsilon_t - \mu}{\sigma}, \quad \eta_t(\boldsymbol{\theta}_h) := \frac{\eta_t - \mu_h}{\sigma_h}, \tag{B.6}$$

are two independent sequences of i.i.d. standard normal random variables. For each $\boldsymbol{\theta}_h \in \boldsymbol{\Theta}_h$, let $(\epsilon, \eta)(\boldsymbol{\theta}_h) := \{(\epsilon_t(\boldsymbol{\theta}_h), \eta_t(\boldsymbol{\theta}_h))\}_{t\in\mathbb{T}}$, which is a sequence of i.i.d. two-dimensional standard normal random vectors under $\mathbb{P}^{\boldsymbol{\theta}_h}$. Then the two-dimensional Gaussian uncertain noises are defined by the family $\{(\epsilon, \eta)(\boldsymbol{\theta}_h) | \boldsymbol{\theta}_h \in \boldsymbol{\Theta}_h\}$ indexed by $\boldsymbol{\Theta}_h$.

Furthermore, under the new probability measure $\mathbb{P}^{\boldsymbol{\theta}_h}$, $\{X_t\}_{t\in\mathbb{T}}$ follows the reference parametric nonlinear time series model:

$$\begin{aligned} X_t &= f(X_{t-1}, X_{t-2}, \cdots, X_{t-p}) + \mu h(X_{t-1}, X_{t-2}, \cdots, X_{t-q}, V_t) \\ &\quad + \sigma h(X_{t-1}, X_{t-2}, \cdots, X_{t-q}, V_t)\epsilon_t(\boldsymbol{\theta}_h), \\ V_t &= f_v(V_{t-1}, V_{t-2}, \cdots, V_{t-p_v}) + \mu_h h_v(V_{t-1}, V_{t-2}, \cdots, V_{t-q_v}) \\ &\quad + \sigma_h h_v(V_{t-1}, V_{t-2}, \cdots, V_{t-q_v})\eta_t(\boldsymbol{\theta}_h). \end{aligned} \tag{B.7}$$

Again, with the "uncertain" parameters $\boldsymbol{\theta}_h := (\mu, \sigma, \mu_h, \sigma_h)$ varying in $\boldsymbol{\Theta}_h$, a family of alternative models in the form of Eq. (B.7) for prediction is defined with respect to the family of probability measures $\{\mathbb{P}^{\boldsymbol{\theta}_h} | \boldsymbol{\theta}_h \in \boldsymbol{\Theta}_h\}$.

To incorporate the family of alternative models in prediction, the conditional sublinear expectation in Eq. (3.8) and the conditional superlinear expectation in Eq. (3.9) with $\boldsymbol{\theta}$ and $\boldsymbol{\Theta}$ replaced by $\boldsymbol{\theta}_h$ and $\boldsymbol{\Theta}_h$, respectively, may be used.

C Bayesian nonlinear expectations under the second model

The Bayesian credible intervals and the Bayesian nonlinear expectations corresponding to the second model in Section 3.2 will be constructed here. From Eq. (B.6), for each $\boldsymbol{\theta}_h \in \boldsymbol{\Theta}_h$,

$$\epsilon_t = \mu + \sigma\epsilon_t(\boldsymbol{\theta}_h), \quad \eta_t = \mu_h + \sigma_h\eta_t(\boldsymbol{\theta}_h), \tag{C.1}$$

where $(\mu, \sigma, \mu_h, \sigma_h)$ are the "uncertain" parameters for the drift and volatility specifications in the observation and latent processes of the reference model in Eq. (3.10). Again, in Bayesian statistics, $(\mu, \sigma, \mu_h, \sigma_h)$ are treated as random variables, and their prior distributions are assigned. Recall from Online Appendix B that under $\mathbb{P}^{\boldsymbol{\theta}_h}$, $\epsilon_1(\boldsymbol{\theta}_h), \epsilon_2(\boldsymbol{\theta}_h), \ldots, \epsilon_T(\boldsymbol{\theta}_h) \overset{i.i.d.}{\sim} N(0,1)$ and $\eta_1(\boldsymbol{\theta}_h), \eta_2(\boldsymbol{\theta}_h), \ldots, \eta_T(\boldsymbol{\theta}_h) \overset{i.i.d.}{\sim} N(0,1)$. Furthermore, $\mathbb{P}^{\boldsymbol{\theta}_h}$, $\{\epsilon_t(\boldsymbol{\theta}_h)\}_{t \in \mathbb{T}}$ and $\{\eta_t(\boldsymbol{\theta}_h)\}_{t \in \mathbb{T}}$ are independent. Consequently, under $\mathbb{P}^{\boldsymbol{\theta}_h}$, $\epsilon_1, \epsilon_2, \ldots, \epsilon_T | (\mu, \sigma) \overset{i.i.d.}{\sim} N(\mu, \sigma^2)$ and $\eta_1, \eta_2, \ldots, \eta_T | (\mu_h, \sigma_h) \overset{i.i.d.}{\sim} N(\mu_h, \sigma_h^2)$. Also, under $\mathbb{P}^{\boldsymbol{\theta}_h}$, given $(\mu, \sigma, \mu_h, \sigma_h)$, $\{\epsilon_t\}_{t \in \mathbb{T}}$ and $\{\eta_t\}_{t \in \mathbb{T}}$ are conditionally independent. As in Section 4, the precisions $\lambda := \frac{1}{\sigma^2}$ and $\lambda_h := \frac{1}{\sigma_h^2}$ are considered, and prior distributions are assigned to (λ, λ_h) instead of (σ, σ_h).

To assign prior distributions for $(\mu, \lambda, \mu_h, \lambda_h)$, for simplicity, it is supposed that (μ, λ) and (μ_h, λ_h) are independent. That is, according to a prior belief, the drift and volatility misspecifications in the observation process are independent of those in the latent process. Again, the prior distributions for (μ, λ) are assigned as the Normal-Gamma prior in Eq. (4.2). Likewise, the prior distributions for (μ_h, λ_h) are assigned as the following Normal-Gamma prior:

$$\mu_h | \lambda_h \sim N\left(\mu_{h0}, \frac{1}{t_{h0}\lambda_h}\right), \quad \lambda_h \sim Ga(\alpha_h, \beta_h), \tag{C.2}$$

where μ_{h0} is the prior mean for μ_h and $t_{h0}\lambda_h$ is the prior precision for $\mu_h|\lambda_h$; α_h and β_h are the prior shape and rate parameters of λ_h, respectively.

Again, the reference model in Eq. (3.10) is estimated using the time series observations $\{X_1, X_2, \ldots, X_n\}$. Given these observations, the latent variables $\{V_1, V_2, \cdots, V_n\}$ are first estimated using (nonlinear) filtering techniques. Suppose $\{\hat{V}_1, \hat{V}_2, \cdots, \hat{V}_n\}$ are the estimated latent variables. Then the quasi-MLE (QMLE) is used to estimate the conditional mean and volatility (f_v, h_v) in the latent process and the conditional mean and volatility (f, h) in the observation process. Further discussions on the estimation of the reference model in Eq. (3.10) and its special case, the SV model, will be discussed in Section 5.1. Now, let $\hat{f}$, $\hat{f}_v$, $\hat{h}$ and $\hat{h}_v$ be the estimates for f, f_v, h and h_v, respectively. Then the residuals $\{e_{vt}\}_{t \in \mathbb{N}}$ and $\{\nu_t\}_{t \in \mathbb{N}}$ from the observation process and the latent process of the reference model in Eq. (3.10) are, respectively, computed as follows:

$$e_{vt} := \frac{X_t - \hat{f}(X_{t-1}, X_{t-2}, \cdots, X_{t-p})}{\hat{h}(X_{t-1}, X_{t-2}, \cdots, X_{t-q}, \hat{V}_t)}, \quad \nu_t := \frac{\hat{V}_t - \hat{f}_v(\hat{V}_{t-1}, \hat{V}_{t-2}, \cdots, \hat{V}_{t-p_v})}{\hat{h}_v(\hat{V}_{t-1}, \hat{V}_{t-2}, \cdots, \hat{V}_{t-q_v})}. \tag{C.3}$$

For each $t \in \mathbb{N}$, the residuals e_{vt} and ν_t are proxies/estimates for the random errors ϵ_t and η_t in the observation and latent processes, respectively. As in Section 4, it is supposed that as an approximation, under $\mathbb{P}^{\boldsymbol{\theta}_h}$, $e_{v1}, e_{v2}, \ldots, e_{vn} | (\mu, \sigma) \overset{i.i.d.}{\sim} N(\mu, \sigma^2)$

and $\nu_1, \nu_2, \ldots, \nu_n | (\mu_h, \sigma_h) \overset{i.i.d.}{\sim} N(\mu_h, \sigma_h^2)$. Also, it is assumed that under $\mathbb{P}^{\boldsymbol{\theta}_h}$, given $(\mu, \sigma, \mu_h, \sigma_h)$, $\{e_{vt}\}_{t\in\mathbb{N}}$ and $\{\nu_t\}_{t\in\mathbb{N}}$ are conditionally independent.

Let $\mathbf{e}_v(n) := (e_{v1}, e_{v2}, \ldots, e_{vn})$. Then as in Section 4, $\lambda|\mathbf{e}_v(n) \sim Ga(\alpha + \frac{n}{2}, \beta_{vn})$, where the posterior shape parameter is $\alpha + \frac{n}{2}$ and the posterior rate parameter β_{vn} is given by:

$$\beta_{vn} := \beta + \frac{1}{2} n s_v^2 + \frac{t_0 n(\mu_0 - \bar{e}_v)^2}{2(t_0 + n)}. \tag{C.4}$$

Here $\bar{e}_v = \frac{1}{n}\sum_{t=1}^{n} e_{vt}$, (i.e., the sample mean of $\mathbf{e}_v(n)$), and $s_v^2 = \frac{1}{n}\sum_{t=1}^{n}(e_{vt} - \bar{e}_v)^2$, (i.e., the sample variance of $\mathbf{e}_v(n)$). Furthermore, the posterior distribution $\mu|\mathbf{e}_v(n) \sim St(\mu_{vn}, \frac{\sigma_{vn}^2}{(t_0+n)}, 2\alpha + n)$, where

$$\mu_{vn} := \frac{t_0\mu_0 + n\bar{e}_v}{t_0 + n}, \tag{C.5}$$

and

$$\sigma_{vn} := \sqrt{\frac{\beta_{vn}}{\alpha + \frac{1}{2}n}}. \tag{C.6}$$

Similarly, let $\boldsymbol{\nu}(n) := (\nu_1, \nu_2, \ldots, \nu_n)$. Then $\lambda_h|\boldsymbol{\nu}(n) \sim Ga(\alpha_h + \frac{n}{2}, \beta_{hn})$, where the posterior shape parameter is $\alpha_h + \frac{n}{2}$ and the posterior rate parameter β_{hn} is given by:

$$\beta_{hn} := \beta_h + \frac{1}{2} n s_h^2 + \frac{t_{h0} n(\mu_{h0} - \bar{\nu})^2}{2(t_{h0} + n)}. \tag{C.7}$$

Here $\bar{\nu} = \frac{1}{n}\sum_{t=1}^{n} \nu_t$, (i.e., the sample mean of $\boldsymbol{\nu}(n)$), and $s_h^2 = \frac{1}{n}\sum_{t=1}^{n}(\nu_t - \bar{\nu})^2$, (i.e., the sample variance of $\boldsymbol{\nu}(n)$).

Furthermore, the posterior distribution $\mu_h|\boldsymbol{\nu}(n) \sim St(\mu_{hn}, \frac{\sigma_{hn}^2}{(t_{h0}+n)}, 2\alpha_h + n)$, where

$$\mu_{hn} := \frac{t_{h0}\mu_{h0} + n\bar{\nu}}{t_{h0} + n}, \tag{C.8}$$

and

$$\sigma_{hn} := \sqrt{\frac{\beta_{hn}}{\alpha_h + \frac{1}{2}n}}. \tag{C.9}$$

A $100(1 - \gamma_1)\%$ Bayesian credible interval for μ given $\mathbf{e}_v(n)$ is $(L_{\mu,v}(\gamma_1), U_{\mu,v}(\gamma_1))$, where

$$L_{\mu,v}(\gamma_1) = \mu_{vn} - t_{2\alpha+n}\left(\frac{\gamma_1}{2}\right)\frac{\sigma_{vn}}{\sqrt{n + t_0}}, \quad U_{\mu,v}(\gamma_1) = \mu_{vn} + t_{2\alpha+n}\left(\frac{\gamma_1}{2}\right)\frac{\sigma_{vn}}{\sqrt{n + t_0}}. \tag{C.10}$$

A $100(1-\gamma_2)\%$ Bayesian credible interval for λ given $\mathbf{e}_v(n)$ is $(L_{\lambda,v}(\gamma_2), U_{\lambda,v}(\gamma_2))$, where

$$L_{\lambda,v}(\gamma_2) = \Gamma_{\alpha+\frac{n}{2},\beta_{vn}}\left(1-\frac{\gamma_2}{2}\right), \quad U_{\lambda,v}(\gamma_2) = \Gamma_{\alpha+\frac{n}{2},\beta_{vn}}\left(\frac{\gamma_2}{2}\right). \tag{C.11}$$

A $100(1-\gamma_{h1})\%$ Bayesian credible interval for μ_h given $\boldsymbol{\nu}(n)$ is $(L_{\mu_h}(\gamma_{h1}), U_{\mu_h}(\gamma_{h1}))$, where

$$L_{\mu_h}(\gamma_{h1}) = \mu_{hn} - t_{2\alpha_h+n}\left(\frac{\gamma_{h1}}{2}\right)\frac{\sigma_{hn}}{\sqrt{n+t_{h0}}}, \quad U_{\mu_h}(\gamma_{h1}) = \mu_{hn} + t_{2\alpha_h+n}\left(\frac{\gamma_{h1}}{2}\right)\frac{\sigma_{hn}}{\sqrt{n+t_{h0}}}. \tag{C.12}$$

A $100(1-\gamma_{h2})\%$ Bayesian credible interval for λ_h given $\boldsymbol{\nu}(n)$ is $(L_{\lambda_h}(\gamma_{h2}), U_{\lambda_h}(\gamma_{h2}))$ where

$$L_{\lambda_h}(\gamma_{h2}) = \Gamma_{\alpha_h+\frac{n}{2},\beta_{hn}}\left(1-\frac{\gamma_{h2}}{2}\right), \quad U_{\lambda_h}(\gamma_{h2}) = \Gamma_{\alpha_h+\frac{n}{2},\beta_{hn}}\left(\frac{\gamma_{h2}}{2}\right). \tag{C.13}$$

Take:

$$\begin{aligned} \mu^- &= \mu_v^-(\gamma_1) = L_{\mu,v}(\gamma_1), \quad \mu^+ = \mu_v^+(\gamma_1) = U_{\mu,v}(\gamma_1), \\ \mu_h^- &= \mu_h^-(\gamma_{h1}) = L_{\mu_h}(\gamma_{h1}), \quad \mu_h^+ = \mu_h^+(\gamma_{h1}) = U_{\mu_h}(\gamma_{h1}), \end{aligned} \tag{C.14}$$

and

$$\begin{aligned} \sigma^- &= \sigma_v^-(\gamma_2) = (U_{\lambda,v}(\gamma_2))^{-\frac{1}{2}}, \quad \sigma^+ = \sigma_v^+(\gamma_2) = (L_{\lambda,v}(\gamma_2))^{-\frac{1}{2}}, \\ \sigma_h^- &= \sigma_h^-(\gamma_{h2}) = (U_{\lambda_h}(\gamma_{h2}))^{-\frac{1}{2}}, \quad \sigma_h^+ = \sigma_h^+(\gamma_{h2}) = (L_{\lambda_h}(\gamma_{h2}))^{-\frac{1}{2}}. \end{aligned} \tag{C.15}$$

Consequently, the product intervals $\mathbf{\Theta}_h$ in Eq. (B.2) are taken as the following product intervals $\mathbf{\Theta}_{\gamma_1,\gamma_2,\gamma_{h1},\gamma_{h2}}(\mathbf{e}_v(n),\boldsymbol{\nu}(n))$ with probability levels γ_1, γ_2, γ_{h1} and γ_{h2}:

$$\begin{aligned} &\mathbf{\Theta}_{\gamma_1,\gamma_2,\gamma_{h1},\gamma_{h2}}(\mathbf{e}(n),\boldsymbol{\nu}(n)) \\ := \; &[\mu_v^-(\gamma_1),\mu_v^+(\gamma_1)] \times [\sigma_v^-(\gamma_2),\sigma_v^+(\gamma_2)] \times [\mu_h^-(\gamma_{h1}),\mu_h^+(\gamma_{h1})] \times [\sigma_h^-(\gamma_{h2}),\sigma_h^+(\gamma_{h2})], \end{aligned} \tag{C.16}$$

and a family of alternative models is defined by the family of probability measures $\{\mathbb{P}(\boldsymbol{\theta}_h)|\boldsymbol{\theta}_h \in \mathbf{\Theta}_{\gamma_1,\gamma_2,\gamma_{h1},\gamma_{h2}}(\mathbf{e}(n),\boldsymbol{\nu}(n))\}$ indexed by $\mathbf{\Theta}_{\gamma_1,\gamma_2,\gamma_{h1},\gamma_{h2}}(\mathbf{e}(n),\boldsymbol{\nu}(n))$.

Then the Bayesian (posterior) sublinear and superlinear expectations for an integrable random variable Y given $\mathcal{F}_n^X$ with respect to $\{\mathbb{P}(\boldsymbol{\theta}_h)|\boldsymbol{\theta}_h \in \mathbf{\Theta}_{\gamma_1,\gamma_2,\gamma_{h1},\gamma_{h2}}(\mathbf{e}(n),\boldsymbol{\nu}(n))\}$ are given by Eq. (4.16) and Eq. (4.17), respectively, with $\boldsymbol{\theta}$ replaced by $\boldsymbol{\theta}_h$ and $\mathbf{\Theta}_{\gamma_1,\gamma_2}(\mathbf{e}(n))$ replaced by $\mathbf{\Theta}_{\gamma_1,\gamma_2,\gamma_{h1},\gamma_{h2}}(\mathbf{e}(n),\boldsymbol{\nu}(n))$.

D Bayesian Shrinkage and Regularization

A (potential) fusion between Bayesian nonlinear expectations and the Bayesian shrinkage and regularization techniques for estimation and variable selection is discussed using the proposed two-stage approach. The key idea of exploring the link between them within the two-stage approach is to first adopt the Bayesian shrinkage and regularization techniques for estimation and variable selection of a reference model in the first stage and then use the Bayesian nonlinear expectations capturing a family of alternative models for prediction in the second stage. The uses of Bayesian shrinkage and regularization techniques for estimation and variable selection have been extensively studied in the literature. See, for example, Carvalho et al. (2009, 2010), Polson and Scott (2010, 2012), Polson et al. (2014), Bhadra et al. (2019), Polson and Sun (2019) and the relevant references therein. Techniques such as the least absolute shrinkage and selection operator (Lasso) and the horseshoe prior are popular techniques for estimation, variable selection and prediction in high-dimensional regression modelling. The Lasso is a regularization technique, while the horseshoe prior is a shrinkage prior. See Bhadra et al. (2019) for the detail and an excellent survey.

In the sequel, the uses of the Lasso and horseshoe prior techniques for the estimation and variable selection of a reference model in the first stage are discussed. Some notations and results in Bhadra et al. (2019) are adopted in the following discussions. To begin with, it is supposed that under the reference probability measure $\mathbb{P}$, the reference model in the first stage is given by a high-dimensional linear regression model with normal random errors:

$$\mathbf{Y} = \mathbf{X}\boldsymbol{\beta} + \bar{\boldsymbol{\epsilon}}, \tag{D.1}$$

where $\mathbf{Y} := (Y_1, Y_2, \ldots, Y_n)' \in \Re^n$, (i.e., a vector of n observations of the response Y; $\boldsymbol{\beta} := (\beta_1, \beta_2, \ldots, \beta_p)' \in \Re^p$, (i.e., a p-dimensional parameter vector); $\bar{\boldsymbol{\epsilon}} := (\bar{\epsilon}_1, \bar{\epsilon}_2, \ldots, \bar{\epsilon}_n)' \in \Re^n$, (i.e., a vector of n random errors). It is assumed that $\bar{\boldsymbol{\epsilon}} \sim N(\mathbf{0}_n, \sigma_{\bar{\epsilon}}\mathbf{I}_n)$, (i.e., a multivariate normal distribution with zero mean vector $\mathbf{0}_n \in \Re^n$ and covariance matrix $\sigma_{\bar{\epsilon}}\mathbf{I}_n$), where $\mathbf{I}_n$ is the $(n \times n)$-identity matrix; $\mathbf{X}$ is the $(n \times p)$ design matrix which consists of n observations of the p covariates/predictors $(X_1, X_2, \cdots, X_p)' \in \Re^p$. To include an intercept in the regression model in Eq. (D.1), we can take $X_1 = 1$, and there are $p - 1$ other covariates/predictors in this case. To describe the situation where the linear regression model in Eq. (D.1) is high-dimensional, it is supposed that $p \gg n$, (i.e., the number of predictors is far more greater than the number of observations).

If the response Y is taken as Y_t and the p predictors $(X_1, X_2, \cdots, X_p)'$ is taken as $(Y_{t-1}, Y_{t-2}, \ldots, Y_{t-p})'$, then the high-dimensional linear regression model in Eq. (D.1) becomes a high-dimensional linear autoregressive time series model with order p.

Sparsity refers to the situation where there are many zeros in the parameter vector $\boldsymbol{\beta}$, (i.e., only a few covariate/predictors are relevant to the prediction of the response Y).

The key to a high-dimensional sparse linear regression model is to identify those non-zero components of $\boldsymbol{\beta}$ and estimate them. Let $\mathbf{X}_i := (X_{1i}, X_{2i}, \ldots, X_{pi})' \in \Re^p$, (i.e., the ith observation of the p predictor), for each $i = 1, 2, \ldots, n$, so that $\mathbf{X} = (\mathbf{X}_1', \mathbf{X}_2', \ldots, \mathbf{X}_n')'$. The Lasso technique is a particular case of a penalized likelihood approach which is described by the following optimization problem:

$$\min_{\boldsymbol{\beta}} \left(\sum_{i=1}^{n} (Y_i - \boldsymbol{\beta}'\mathbf{X}_i)^2 + \text{pen}_\kappa(\boldsymbol{\beta}) \right), \tag{D.2}$$

where $\text{pen}_\kappa(\boldsymbol{\beta})$ is the following separable penalty function:

$$\text{pen}_\kappa(\boldsymbol{\beta}) := \sum_{j=1}^{p} p_\kappa(\beta_j). \tag{D.3}$$

Note that $p_\kappa(\beta_j)$ is interpreted as the penalty on the parameter β_j, for each $j = 1, 2, \ldots, p$. If the individual penalty $p_\kappa(\beta_j)$ in Eq. (D.3) is taken as $\kappa|\beta_j|$, where $\kappa > 0$, then the optimization problem in Eq. (D.2) will result in the Lasso, (i.e., the Lasso corresponds to the situation where the penalty is given by the l_1-norm. The key idea of the Lasso is to perform estimation and variable selection simultaneously in the sense that it gives rise to a "true" sparse solution with some of the estimates being zero. The sum of squared errors $\sum_{i=1}^{n}(Y_i - \boldsymbol{\beta}'\mathbf{X}_i)^2$ is a measure of fit, which is related to the negative of a log-likelihood function, say $l(\boldsymbol{\beta}|\mathbf{Y}, \mathbf{X})$, where $(\mathbf{Y}, \mathbf{X})$ represents the given data.

The horseshoe prior is a global-local shrinkage prior, which is assigned to the regression coefficients β_i in the parameter vector $\boldsymbol{\theta}$ under a Bayesian hierarchical linear regression model with $p \leqslant n$:

$$\begin{aligned} &\mathbf{Y} = \mathbf{X}\boldsymbol{\beta} + \bar{\boldsymbol{\epsilon}}, \quad \bar{\boldsymbol{\epsilon}} \sim N(\mathbf{0}_n, \sigma_{\bar{\epsilon}}\mathbf{I}_n), \\ &\beta_j|\kappa_j, \tau, \sigma_{\bar{\epsilon}} \sim N(0, \kappa_j^2\tau^2\sigma_{\bar{\epsilon}}^2), \\ &\kappa_j \sim f_{\kappa_j}(\cdot), \quad \tau \sim g_\tau(\cdot), \quad \sigma_{\bar{\epsilon}} \sim h_{\sigma_{\bar{\epsilon}}}(\cdot). \end{aligned} \tag{D.4}$$

Note that τ may be interpreted as a global shrinkage prior and that for each $j = 1, 2, \ldots, p$, κ_j may be interpreted as a local shrinkage prior for the regression coefficient β_j. Intuitively, if τ becomes smaller, all the regression coefficients shrink to zero. If κ_j becomes smaller, the regression coefficient β_j shrinks to zero.

When the sum of squared errors $\sum_{i=1}^{n}(Y_i - \boldsymbol{\beta}'\mathbf{X}_i)^2$ is replaced with the negative of a log-likelihood function, say $l(\boldsymbol{\beta}|\mathbf{Y}, \mathbf{X})$, the optimization problem in Eq. (D.2) becomes:

$$\min_{\boldsymbol{\beta}} \left(l(\boldsymbol{\beta}|\mathbf{Y}, \mathbf{X}) + \text{pen}_\kappa(\boldsymbol{\beta}) \right). \tag{D.5}$$

The regularization in Eq. (D.5) is closely linked with a Bayesian probabilistic approach resulting in a Bayesian hierarchical model:

$$\begin{aligned} p(\boldsymbol{\beta}|\mathbf{Y},\mathbf{X}) &\propto p(\mathbf{Y},\mathbf{X}|\boldsymbol{\beta})\pi_\kappa(\boldsymbol{\beta}) \\ &\propto \exp(-l(\boldsymbol{\beta}|\mathbf{Y},\mathbf{X})\exp(-\mathrm{pen}_\kappa(\boldsymbol{\beta})), \end{aligned} \tag{D.6}$$

where $p(\boldsymbol{\beta}|\mathbf{Y},\mathbf{X})$ is the posterior density of $\boldsymbol{\beta}$ given the data $(\mathbf{Y},\mathbf{X})$; $p(\mathbf{Y},\mathbf{X}|\boldsymbol{\beta})$ is the likelihood function of $\boldsymbol{\beta}$; $\pi_\kappa(\boldsymbol{\beta})$ is the prior density of $\boldsymbol{\beta}$. It can be seen that the solution to the optimization problem in Eq. (D.5) coincides with the posterior mode maximizing the posterior density $p(\boldsymbol{\beta}|\mathbf{Y},\mathbf{X})$ in Eq. (D.6). That is,

$$\hat{\boldsymbol{\beta}} := \arg - \max_{\boldsymbol{\beta}} p(\boldsymbol{\beta}|\mathbf{Y},\mathbf{X}). \tag{D.7}$$

Also, the common standard deviation $\sigma_{\bar{\epsilon}}$ of the random errors is estimated by:

$$\hat{\sigma}_{\bar{\epsilon}} := \sqrt{\frac{\sum_{i=1}^n (Y_i - \hat{\boldsymbol{\beta}}'\mathbf{X}_i)^2}{n-p}}. \tag{D.8}$$

Then Eq. (D.7) and Eq. (D.8) give rise to the results for the estimation and variable selection of the reference model in Eq. (D.1) in the first stage.

To proceed with the second stage, a family of alternative models is defined. To define the family of alternative models, a family of probability measures $\{\mathbb{P}^{\boldsymbol{\theta}}|\boldsymbol{\theta} \in \boldsymbol{\Theta}\}$ equivalent to the reference probability measure $\mathbb{P}$ is specified using the discrete-time Girsanov's transform as in Section 3.1. Specifically, for each $\boldsymbol{\theta} \in \boldsymbol{\Theta}$, under the probability measure $\mathbb{P}^{\boldsymbol{\theta}}$, the data follow the linear regression model:

$$Y_i = \boldsymbol{\beta}'\mathbf{X}_i + \mu\sigma_{\bar{\epsilon}} + \sigma\bar{\epsilon}_i(\boldsymbol{\theta}), \quad i \in \mathbb{T}, \tag{D.9}$$

where (μ, σ) are the "uncertain" parameters for the conditional mean and volatility misspecifications; $\{\bar{\epsilon}_i(\boldsymbol{\theta})\}_{i\in\mathbb{T}}$ is a sequence of i.i.d. normal random variables with mean 0 and variance $\sigma^2_{\bar{\epsilon}}$ under $\mathbb{P}^{\boldsymbol{\theta}}$ with $\bar{\epsilon}_i(\boldsymbol{\theta}) := \frac{\bar{\epsilon}_t - \mu}{\sigma}$.

Given the data $(\mathbf{Y},\mathbf{X})$ the residuals from the reference model are computed as follows:

$$\bar{e}_i = \frac{Y_i - \hat{\boldsymbol{\beta}}'\mathbf{X}_i}{\hat{\sigma}_{\bar{\epsilon}}}, \quad i = 1, 2, \ldots, n, \tag{D.10}$$

where $\hat{\boldsymbol{\beta}}$ is given by Eq. (D.7) and $\hat{\sigma}_{\bar{\epsilon}}$ is given by Eq. (D.8).

Let $\bar{\mathbf{e}}(n) := (\bar{e}_1, \bar{e}_2, \ldots, \bar{e}_n)$, (i.e., the n observations of the residuals from the reference model). Then the Bayesian credible intervals for the uncertain parameters (μ, σ) given $\bar{\mathbf{e}}(n)$ can be computed by following the same procedures as in Section 4, and a family of

alternative models for evaluating the Bayesian nonlinear expectations can be defined by specifying a family of probability measures $\{\mathbb{P}^{\boldsymbol{\theta}}|\boldsymbol{\theta} \in \boldsymbol{\Theta}_{\gamma_1,\gamma_2}(\bar{\mathrm{e}}(n))\}$ indexed by $\boldsymbol{\Theta}_{\gamma_1,\gamma_2}(\bar{\mathrm{e}}(n))$ accordingly.

Let $\mathbb{F}^Y$ be the filtration $\{\mathcal{F}_t^Y\}_{t\in\mathbb{T}}$, where $\mathcal{F}_t^Y$ is the $\mathbb{P}$-completed σ-field generated by $\{Y_1, Y_2, \ldots, Y_t\}$, (i.e., the information about the t observations of the response Y). Also, $\mathbb{F}^{\mathbf{X}}$ be the filtration $\{\mathcal{F}_t^{\mathbf{X}}\}_{t\in\mathbb{T}}$, where $\mathcal{F}_t^{\mathbf{X}}$ is the $\mathbb{P}$-completed σ-field generated by $\{\mathbf{X}_1, \mathbf{X}_2, \ldots, \mathbf{X}_t\}$, (i.e., the information about the t observations of the p predictors). Then a Bayesian (posterior) sublinear expectation for Y_{n+1} given $\mathcal{F}_n^Y \vee \mathcal{F}_{n+1}^{\mathbf{X}}$, (i.e., the minimal σ-field containing the σ-fields $\mathcal{F}_n^Y$ and $\mathcal{F}_{n+1}^{\mathbf{X}}$), with respect to $\{\mathbb{P}(\boldsymbol{\theta})|\boldsymbol{\theta} \in \boldsymbol{\Theta}_{\gamma_1,\gamma_2}(\bar{\mathrm{e}}(n))\}$ is given by:

$$\mathbb{CBE}_{\boldsymbol{\Theta}_{\gamma_1,\gamma_2}(\bar{\mathrm{e}}(n))}[Y_{n+1}|\mathcal{F}_n^Y \vee \mathcal{F}_{n+1}^{\mathbf{X}}] := \text{ess} - \sup_{\boldsymbol{\theta}\in\boldsymbol{\Theta}_{\gamma_1,\gamma_2}(\bar{\mathrm{e}}(n))} \mathrm{E}^{\boldsymbol{\theta}}[Y_{n+1}|\mathcal{F}_n^Y \vee \mathcal{F}_{n+1}^{\mathbf{X}}]. \quad \text{(D.11)}$$

Similarly, a Bayesian (posterior) superlinear expectation for Y_{n+1} given $\mathcal{F}_n^Y \vee \mathcal{F}_{n+1}^{\mathbf{X}}$ with respect to $\{\mathbb{P}(\boldsymbol{\theta})|\boldsymbol{\theta} \in \boldsymbol{\Theta}_{\gamma_1,\gamma_2}(\bar{\mathrm{e}}(n))\}$ is given by:

$$\mathbb{CBIE}_{\boldsymbol{\Theta}_{\gamma_1,\gamma_2}(\bar{\mathrm{e}}(n))}[Y_{n+1}|\mathcal{F}_n^Y \vee \mathcal{F}_{n+1}^{\mathbf{X}}] := \text{ess} - \inf_{\boldsymbol{\theta}\in\boldsymbol{\Theta}_{\gamma_1,\gamma_2}(\bar{\mathrm{e}}(n))} \mathrm{E}^{\boldsymbol{\theta}}[Y_{n+1}|\mathcal{F}_n^Y \vee \mathcal{F}_{n+1}^{\mathbf{X}}]. \quad \text{(D.12)}$$

One may further explore the link between the Bayesian nonlinear expectations and the Bayesian shrinkage and regularization techniques. The current paper focuses on the implementation of some cases of the nonlinear autoregressive model in Eq. (3.1) and the product process in Eq. (3.10).

E Forecasting

The upper, lower and interval forecasts as well as the Bayesian risk-neutral forecasts under the two reference models in Eq. (3.1) and Eq. (3.10) are derived here. Firstly, the reference model in Eq. (3.1) is considered. Let $\{X_t\}_{t\in\mathbb{T}}$ be the daily percentage logarithmic returns of a financial asset. An upper point forecast for X_{n+1} given information $\mathcal{F}_n^X$ based on a Bayesian posterior sublinear expectation with respect to $\{\mathbb{P}^{\boldsymbol{\theta}}|\boldsymbol{\theta} \in \boldsymbol{\Theta}_{2\gamma_1,2\gamma_2}(\mathrm{e}(n))\}$ [1] is defined as:

$$\mathbb{CBE}_{\boldsymbol{\Theta}_{2\gamma_1,2\gamma_2}(\mathrm{e}(n))}[X_{n+1}|\mathcal{F}_n^X] := \text{ess} - \sup_{\boldsymbol{\theta}\in\boldsymbol{\Theta}_{2\gamma_1,2\gamma_2}(\mathrm{e}(n))} \mathrm{E}^{\boldsymbol{\theta}}[X_{n+1}|\mathcal{F}_n^X]. \quad \text{(E.1)}$$

[1]Note that the space $\boldsymbol{\Theta}_{\gamma_1,\gamma_2}(\mathrm{e}(n))$ is specified by the two-sided Bayesian credible intervals for the "uncertain" parameters (μ, σ) as in Eq (4.15); however, one-sided critical values are used for the computation of the upper-point forecast. To take account of this, $\{\mathbb{P}^{\boldsymbol{\theta}}|\boldsymbol{\theta} \in \boldsymbol{\Theta}_{2\gamma_1,2\gamma_2}(\mathrm{e}(n))\}$ instead of $\{\mathbb{P}^{\boldsymbol{\theta}}|\boldsymbol{\theta} \in \boldsymbol{\Theta}_{\gamma_1,\gamma_2}(\mathrm{e}(n))\}$ is adopted to compute the upper-point forecast. The same convention applies when one-sided critical values are used to compute other forecasts and risk metrics to be presented in the sequel.

Similarly, a lower-point forecast for X_{n+1} given information $\mathcal{F}_n^X$ based on a Bayesian posterior superlinear expectation with respect to $\{\mathbb{P}^{\boldsymbol{\theta}}|\boldsymbol{\theta} \in \boldsymbol{\Theta}_{2\gamma_1,2\gamma_2}(\mathbf{e}(n))\}$ is defined as:

$$\mathbb{CBIE}_{\boldsymbol{\Theta}_{2\gamma_1,2\gamma_2}(\mathbf{e}(n))}[X_{n+1}|\mathcal{F}_n^X] := \text{ess} - \inf_{\boldsymbol{\theta}\in\boldsymbol{\Theta}_{2\gamma_1,2\gamma_2}(\mathbf{e}(n))} \mathrm{E}^{\boldsymbol{\theta}}[X_{n+1}|\mathcal{F}_n^X]. \tag{E.2}$$

From Eq. (3.7), the upper-point forecast with respect to $\{\mathbb{P}^{\boldsymbol{\theta}}|\boldsymbol{\theta} \in \boldsymbol{\Theta}_{2\gamma_1,2\gamma_2}(\mathbf{e}(n))\}$ is given by:

$$\begin{aligned} &\mathbb{CBE}_{\boldsymbol{\Theta}_{2\gamma_1,2\gamma_2}(\mathbf{e}(n))}[X_{n+1}|\mathcal{F}_n^X] \\ = \; &\hat{f}(X_n, X_{n-1}, \cdots, X_{n+1-p}) + U_\mu(2\gamma_1)\hat{g}(X_n, X_{n-1}, \cdots, X_{n+1-q}), \end{aligned} \tag{E.3}$$

and the lower-point forecast with respect to $\{\mathbb{P}^{\boldsymbol{\theta}}|\boldsymbol{\theta} \in \boldsymbol{\Theta}_{2\gamma_1,2\gamma_2}(\mathbf{e}(n))\}$ is given by:

$$\begin{aligned} &\mathbb{CBIE}_{\boldsymbol{\Theta}_{2\gamma_1,2\gamma_2}(\mathbf{e}(n))}[X_{n+1}|\mathcal{F}_n^X] \\ = \; &\hat{f}(X_n, X_{n-1}, \cdots, X_{n+1-p}) + L_\mu(2\gamma_1)\hat{g}(X_n, X_{n-1}, \cdots, X_{n+1-q}), \end{aligned} \tag{E.4}$$

where $L_\mu(2\gamma_1)$ and $U_\mu(2\gamma_1)$ are, respectively, given by Eq. (4.9) and Eq. (4.10) with γ_1 replaced by $2\gamma_1$; $\hat{f} := \hat{f}(X_n, X_{n-1}, \cdots, X_{n+1-p})$ and $\hat{g} := \hat{g}(X_n, X_{n-1}, \cdots, X_{n+1-q})$ are the estimated conditional mean and volatility of the reference model in Eq. (3.1), respectively.

Then an interval forecast based on the Bayesian sublinear and superlinear expectations with respect to $\{\mathbb{P}^{\boldsymbol{\theta}}|\boldsymbol{\theta} \in \boldsymbol{\Theta}_{\gamma_1,\gamma_2}(\mathbf{e}(n))\}$ is given by:

$$\begin{aligned} &(\mathbb{CBIE}_{\boldsymbol{\Theta}_{\gamma_1,\gamma_2}(\mathbf{e}(n))}[X_{n+1}|\mathcal{F}_n^X], \mathbb{CBE}_{\boldsymbol{\Theta}_{\gamma_1,\gamma_2}(\mathbf{e}(n))}[X_{n+1}|\mathcal{F}_n^X]) \\ = \; &(\hat{f} + L_\mu(\gamma_1)\hat{g}, \hat{f} + U_\mu(\gamma_1)\hat{g}). \end{aligned} \tag{E.5}$$

For comparison, a Bayesian risk-neutral forecast for X_{n+1} given $\mathcal{F}_n^X$ may also be considered, and it is given by:

$$\mathbb{BRNF}[X_{n+1}|\mathcal{F}_n^X] = \hat{f}(X_n, X_{n-1}, \cdots, X_{n+1-p}) + \mu_n\hat{g}(X_n, X_{n-1}, \cdots, X_{n+1-q}), \tag{E.6}$$

where μ_n is given by Eq. (4.6).

Now the reference model in Eq. (3.10) is considered. An upper-point (lower-point) forecast for X_{n+1} given $\mathcal{F}_n^X$ based on a Bayesian posterior sublinear (superlinear) expectation with respect to $\{\mathbb{P}(\boldsymbol{\theta}_h)|\boldsymbol{\theta}_h \in \boldsymbol{\Theta}_{2\gamma_1,2\gamma_2,2\gamma_{h1},2\gamma_{h2}}(\mathbf{e}(n), \boldsymbol{\nu}(n))\}$ is given by Eq. (E.1) (Eq. (E.2)) with $\boldsymbol{\theta}$ replaced by $\boldsymbol{\theta}_h$ and $\boldsymbol{\Theta}_{2\gamma_1,2\gamma_2}(\mathbf{e}(n))$ replaced by $\boldsymbol{\Theta}_{2\gamma_1,2\gamma_2,2\gamma_{h1},2\gamma_{h2}}(\mathbf{e}(n), \boldsymbol{\nu}(n))$. From Eq. (B.7), the upper-point forecast is given by:

$$\begin{aligned} &\mathbb{CBE}_{\boldsymbol{\Theta}_{2\gamma_1,2\gamma_2,2\gamma_{h1},2\gamma_{h2}}(\mathbf{e}(n),\boldsymbol{\nu}(n))}[X_{n+1}|\mathcal{F}_n^X] = \hat{f}(X_n, X_{n-1}, \cdots, X_{n+1-p}) \\ &+U_{\mu,v}(2\gamma_1) \max_{\mu_h\in[L_{\mu_h}(2\gamma_{h1}),U_{\mu_h}(2\gamma_{h1})]} \hat{h}(X_n, X_{n-1}, \cdots, X_{n+1-q}, \hat{V}_{n+1}(\mu_h)), \end{aligned} \tag{E.7}$$

where $\hat{f} := \hat{f}(X_n, X_{n-1}, \cdots, X_{n+1-p})$ and $\hat{h}(\mu_h) := \hat{h}(X_n, X_{n-1}, \cdots, X_{n+1-q}, \hat{V}_{n+1}(\mu_h))$ are the estimated conditional mean and volatility in the observation process of the reference model in Eq. (3.10), respectively; $U_{\mu,v}(2\gamma_1)$ is given by Eq. (C.10) with γ_1 replaced by $2\gamma_1$; $L_{\mu_h}(2\gamma_{h1})$ and $U_{\mu_h}(2\gamma_{h1})$ are given by Eq. (C.12) with γ_{h1} replaced by $2\gamma_{h1}$; $\hat{V}_{n+1}(\mu_h)$ is given by:

$$\hat{V}_{n+1}(\mu_h) = \hat{f}_v(\hat{V}_n, \hat{V}_{n-1}, \cdots, \hat{V}_{n+1-p_v}) + \mu_h \hat{h}_v(\hat{V}_n, \hat{V}_{n-1}, \cdots, \hat{V}_{n+1-q_v}), \tag{E.8}$$

where $\hat{f}_v := \hat{f}_v(\hat{V}_n, \hat{V}_{n-1}, \cdots, \hat{V}_{n+1-p_v})$ and $\hat{h}_v := \hat{h}_v(\hat{V}_n, \hat{V}_{n-1}, \cdots, \hat{V}_{n+1-q_v})$ are the estimated conditional mean and volatility in the latent process of the reference model in Eq. (3.10), respectively; $\{\hat{V}_t\}_{t\in\mathbb{N}}$ is the estimated latent process from (nonlinear) filtering given the observations $\{X_t\}_{t\in\mathbb{N}}$.

Similarly, the lower-point forecast is given by:

$$\begin{aligned} &\mathbb{CBIE}_{\boldsymbol{\Theta}_{2\gamma_1,2\gamma_2,2\gamma_{h1},2\gamma_{h2}}(\mathbf{e}(n),\boldsymbol{\nu}(n))}[X_{n+1}|\mathcal{F}_n^X] = \hat{f}(X_n, X_{n-1}, \cdots, X_{n+1-p}) \\ &+L_{\mu,v}(2\gamma_1) \min_{\mu_h \in [L_{\mu_h}(2\gamma_{h1}), U_{\mu_h}(2\gamma_{h1})]} \hat{h}(X_n, X_{n-1}, \cdots, X_{n+1-q}, \hat{V}_{n+1}(\mu_h)), \end{aligned} \tag{E.9}$$

where $L_{\mu,v}(2\gamma_1)$ is given by Eq. (C.10) with γ_1 replaced by $2\gamma_1$ and $\hat{V}_{n+1}(\mu_h)$ is given by Eq. (E.8).

An interval estimate with respect to $\{\mathbb{P}(\boldsymbol{\theta}_h)|\boldsymbol{\theta}_h \in \boldsymbol{\Theta}_{\gamma_1,\gamma_2,\gamma_{h1},\gamma_{h2}}(\mathbf{e}(n), \boldsymbol{\nu}(n))\}$ is:

$$\begin{aligned} &(\mathbb{CBIE}_{\boldsymbol{\Theta}_{\gamma_1,\gamma_2,\gamma_{h1},\gamma_{h2}}(\mathbf{e}(n),\boldsymbol{\nu}(n))}[X_{n+1}|\mathcal{F}_n^X], \mathbb{CBE}_{\boldsymbol{\Theta}_{\gamma_1,\gamma_2,\gamma_{h1},\gamma_{h2}}(\mathbf{e}(n),\boldsymbol{\nu}(n))}[X_{n+1}|\mathcal{F}_n^X]) \\ = \; &(\hat{f} + L_{\mu,v}(\gamma_1) \min_{\mu_h \in [L_{\mu_h}(\gamma_{h1}), U_{\mu_h}(\gamma_{h1})]} \hat{h}(\mu_h), \hat{f} + U_{\mu,v}(\gamma_1) \max_{\mu_h \in [L_{\mu_h}(\gamma_{h1}), U_{\mu_h}(\gamma_{h1})]} \hat{h}(\mu_h)). \end{aligned} \tag{E.10}$$

A Bayesian risk-neutral forecast for X_{n+1} given $\mathcal{F}_n^X$ is:

$$\begin{aligned} &\mathbb{BRNF}_h[X_{n+1}|\mathcal{F}_n^X] \\ = \; &\hat{f}(X_n, X_{n-1}, \cdots, X_{n+1-p}) + \mu_{vn} \hat{h}(X_n, X_{n-1}, \cdots, X_{n+1-q}, \hat{V}_{n+1}(\mu_{hn})), \end{aligned} \tag{E.11}$$

where μ_{vn} and μ_{hn} are given by Eq. (C.5) and Eq. (C.8), respectively.

When the reference model is the first-order SV model in Eq. (3.12), the estimated latent process $\{\hat{V}_t\}_{t\in\mathbb{N}}$ is obtained by the Kalman filter, and the estimates $(\hat{\alpha}_v, \hat{\delta}_v, \hat{\sigma}_v)$ for the unknown parameters $(\alpha_v, \delta_v, \sigma_v)$ are obtained from the QMLE. In this case, $\hat{V}_{n+1}(\mu_h)$ in Eq. (E.8) becomes:

$$\hat{V}_{n+1}(\mu_h) = \hat{\alpha}_v + \mu_h \hat{\sigma}_v + \hat{\delta}_v \hat{V}_n. \tag{E.12}$$

Then the upper-point forecast in Eq. (E.7) becomes:

$$\mathbb{CBE}_{\mathbf{\Theta}_{2\gamma_1,2\gamma_2,2\gamma_{h1},2\gamma_{h2}}(\mathbf{e}(n),\boldsymbol{\nu}(n))}[X_{n+1}|\mathcal{F}_n^X] = U_{\mu,v}(2\gamma_1)\exp(\hat{V}_{n+1}(U_{\mu_h}(2\gamma_{h1}))/2), \tag{E.13}$$

and the lower-point forecast in Eq. (E.9) becomes:

$$\mathbb{CBIE}_{\mathbf{\Theta}_{2\gamma_1,2\gamma_2,2\gamma_{h1},2\gamma_{h2}}(\mathbf{e}(n),\boldsymbol{\nu}(n))}[X_{n+1}|\mathcal{F}_n^X] = L_{\mu,v}(2\gamma_1)\exp(\hat{V}_{n+1}(L_{\mu_h}(2\gamma_{h1}))/2). \tag{E.14}$$

An interval estimate in Eq. (E.10) becomes:

$$\begin{aligned} & (\mathbb{CBIE}_{\mathbf{\Theta}_{\gamma_1,\gamma_2,\gamma_{h1},\gamma_{h2}}(\mathbf{e}(n),\boldsymbol{\nu}(n))}[X_{n+1}|\mathcal{F}_n^X], \mathbb{CBE}_{\mathbf{\Theta}_{\gamma_1,\gamma_2,\gamma_{h1},\gamma_{h2}}(\mathbf{e}(n),\boldsymbol{\nu}(n))}[X_{n+1}|\mathcal{F}_n^X]) \\ = \ & (L_{\mu,v}(\gamma_1)\exp(\hat{V}_{n+1}(L_{\mu_h}(\gamma_{h1}))/2), U_{\mu,v}(\gamma_1)\exp(\hat{V}_{n+1}(U_{\mu_h}(\gamma_{h1}))/2)). \end{aligned} \tag{E.15}$$

The Bayesian risk-neutral forecast in Eq. (E.11) becomes:

$$\mathbb{BRNF}_h[X_{n+1}|\mathcal{F}_n^X] = \mu_{vn}\exp(\hat{V}_{n+1}(\mu_{hn})/2). \tag{E.16}$$

F Some Potential Generalizations

Some preliminary discussions on potential extensions of the proposed two-stage approach to nonparametric time series modelling are provided at an intuitive level. Further studies on these potential extensions may be considered as potential topics for future research. Since a nonparametric approach does not impose stringent assumptions for the parametric form of a model, it may reduce prejudice in the specification of the model. Consequently, it may be expected that misspecifications in the conditional mean and volatility of a nonlinear time series model may be reduced if a nonparametric approach is adopted to estimate the model. However, one may still consider the use of the proposed two-stage approach when the nonparametric approach is used for at least two reasons. Firstly, though misspecifications in the conditional mean and volatility may be reduced, they may not be completely eliminated. Secondly, it may be possible that future developments may deviate from an estimated model using the past and the current data. Consequently, it may still be relevant to consider a family of alternative models for prediction.

To discuss the possibility of using a nonparametric approach in the two-stage approach, the estimation of the conditional mean and volatility functions of the reference model in Eq. (3.1) in the first stage is firstly considered. The estimation of the reference model in Eq. (3.10) using a nonparametric approach is more complicated since it

involves a latent process and the nonparametric approach for filtering the latent process is complicated. There are some nonparemtric statistical methods for the estimation and variable selection of nonlinear time series models in the literature. See, for example, Fan and Yao (2003), Chapter 8, and the relevant references therein. To estimate the conditional mean of the reference model in Eq. (3.1), a local regression, such as a local linear regression or a local quadratic regression, may be used. The basic idea of a local regression is to first use the Taylor's expansion to approximate the conditional mean function and then use the local least-square method to estimate the approximating function. The key is to select an optimal bandwidth for smoothing and a kernel function for locally weighting. See Fan and Yao (2003), Chapter 8, Sections 8.3-8.5, for the detail. To estimate the conditional volatility function of the reference model in Eq. (3.1), the squared residuals $\{r_t^2\}_{t \in \mathbb{N}}$ are used, where

$$r_t := X_t - \hat{f}(X_{t-1}, X_{t-2}, \dots, X_{t-p}), \tag{F.1}$$

where $\hat{f}$ is an estimate for the conditional mean function f based on the local regression. Then the nonparametric regression of r_t^2 on $X_{t-1}, X_{t-2}, \dots, X_{t-p}$ coupled with the local regression techniques are used to estimate the volatility function g as in the estimation of the conditional mean. An alternative method is to use the local pseudolikelihood method based on the assumption that the random error $\epsilon_t \sim N(0, 1)$. See Fan and Yao (2003), Chapter 8, Section 8.7, for the detail.

After estimating the conditional mean and volatility functions of the reference model in Eq. (3.1), at the second stage, the residuals from the estimated reference model can be computed, and the Bayesian credible intervals for the drift and volatility misspecifications and the Bayesian nonlinear predictions can be constructed as in Section 4. It may be recalled the assumption that the residuals $\{e_1, e_2, \dots, e_n\}$ from the estimated reference model are conditionally i.i.d. standard normal random variables given the "uncertain" parameters (μ, σ) under a probability measure $\mathbb{P}^{\boldsymbol{\theta}}$ defined by a discrete-time Girsanov's transform. The advantage of imposing this assumption is that closed-form Bayesian credible intervals for the "uncertain" parameters (μ, σ) are obtained using conjugate priors. However, one may explore the possibility of relaxing this parametric assumption and consider a Bayesian nonparametric approach to construct the Bayesian credible intervals which are used for defining a family of alternative models underlying the Bayesian nonlinear expectations for prediction. Specifically, one may consider using the Bayesian nonparametric density estimation in Lo (1984) to construct the Bayesian credible intervals, where a random density function was introduced by convoluting a given kernel with a Dirichlet process. Furthermore, one may explore the possibility of using a Bayesian nonparametric approach to estimate the reference model in the first stage and to construct the Bayesian credible intervals in the second stage. It seems that the paper by Lau and Siu (2008) might provide a clue for exploring an extension along this line.

It may be noted that in the reference models in Eq. (3.1) and Eq. (3.10), the focus is

to modelling the conditional mean and variance. This focus is relevant to applications. Tong (1990), see Page 98 therein, noted that the formulation of a nonlinear autoregressive model with the conditional mean and volatility functions may be compared with a continuous-time diffusion process. Indeed, a continuous-time diffusion process has important applications in economics, finance and actuarial science and may also have interesting applications in biological and physical sciences. Fan and Yao (2003), see Chapter 8 therein, focused on the estimation of the conditional mean and volatility functions when discussing the nonparametric approach to estimate nonlinear time series models. They also pointed out that the conditional mean is optimal for prediction and the conditional volatility is used for constructing confidence intervals and testing statistical hypotheses. As far as applications in finance and financial econometrics are concerned, the conditional mean and volatility functions play a significant role in asset pricing, mean-variance portfolio selection and risk analysis. For Bitcoin applications, it will be seen in Section 6 that the empirical Bitcoin data reveals that the trend and volatility of the Bitcoin series change dramatically in some periods of Covid 19. This may indicate that modelling and predicting the conditional mean and volatility functions of the Bitcoin series are practically relevant and challenging. Besides Bitcoin applications, the proposed two-stage approach may also be applied to long-term actuarial forecasting and stochastic mortality modelling where the conditional mean and volatility functions may also play an important role.

G Results on different sets of prior parameters

Tables G.1-G.3 below give the 95% BCIs and BRNEs for the "uncertain" parameters $(\mu, \sigma, \mu_h, \sigma_h)$, the one-step-ahead forecasts for the Bitcoin return, the one-step-ahead VaR and ES predictions for the short position of one unit of the Bitcoin, respectively, from the SETAR(2,1,1) model, the GARCH(1,1) model and the first-order SV model under the prior parameters in Set 2. Tables G.4-G.6 present the respective results under the prior parameters in Set 3.

~ Tables G.1-G.6 about here ~

From Tables G.1-G.6, it may be seen that the results under the prior parameters in Set 2 and Set 3 are qualitatively similar to those under the prior parameters in Set 1.

Table G.1: 95% BCIs and BRNEs for μ, σ, μ_h and σ_h (Set 2 priors)

	SETAR	GARCH	SV
95% BCI for μ	(-0.1699211, 0.1699211)	(-0.02640707, 0.0589137)	(-0.04661844, 0.08682969)
BRNE for μ	1.046902e-16	0.01625332	0.02010563
95% BCI for σ	(3.869872, 4.110908)	(0.9715702, 1.032084)	(1.519609, 1.614258)
BRNE for σ	3.98646	1.000841	1.56539
95% BCI for μ_h	-	-	(0.001556987, 0.004768124)
BRNE for μ_h	-	-	0.003162555
95% BCI for σ_h	-	-	(0.03765428, 0.03999794)
BRNE σ_h	-	-	0.03878793

Table G.2: One-step-ahead forecasts (Set 2 priors)

	SETAR	GARCH	SV
LPF	0.1625994	-0.0408636	-0.1084622
UPF	0.5024416	0.09928537	0.2020942
IF	(0.1300082, 0.5350327)	(-0.05430401, 0.1127258)	(-0.1382325, 0.2318892)
BRNF	0.3325205	0.02921088	0.04678652
CF	0.3325205	0	0

Table G.3: Estimates of VaR and ES (Set 2 priors)

	SETAR	GARCH	SV
UVaR	2825.056	1123.42	2481.96
UES	3493.095	1399.006	3092.513
LVaR	2538.708	1005.332	2219.412
LES	3167.578	1264.764	2793.947
IVaR	(2512.058, 2853.336)	(994.3418, 1135.083)	(2194.984, 2507.898)
IES	(3137.378, 3525.34)	(1252.31, 1412.303)	(2766.256, 3122.096)
BRNVaR	2679.368	1063.34	2348.364
BRNES	3327.184	1330.585	2940.321
VaR	768.9944	1049.765	1487.999
ES	931.4985	1316.447	1866.011

Table G.4: 95% BCIs and BRNEs for μ, σ, μ_h and σ_h (Set 3 priors)

	SETAR	GARCH	SV
95% BCI for μ	(-0.1334772, 0.1967283)	(0.005193223, 0.08871404)	(-0.0144201, 0.1155934)
BRNE for μ	0.03162555	0.04695363	0.05058665
95% BCI for σ	(3.871959, 4.113124)	(0.9793575, 1.040357)	(1.524526, 1.619482)
BRNE for σ	3.98861	1.008863	1.570456
95% BCI for μ_h	-	-	(0.02620631, 0.0370448)
BRNE for μ_h	-	-	0.03162555
95% BCI for σ_h	-	-	(0.1270912, 0.1350071)
BRNE σ_h	-	-	0.1309201

Table G.5: One-step-ahead forecasts (Set 3 priors)

	SETAR	GARCH	SV
LPF	0.1990433	0.01082386	-0.03364754
UPF	0.5292487	0.148009	0.2700684
IF	(0.1673763, 0.5609158)	(-0.002332311, 0.1611651)	(-0.06273335, 0.2992359)
BRNF	0.364146	0.07941642	0.1181133
CF	0.3325205	0	0

Table G.6: Estimates of VaR and ES (Set 3 priors)

	SETAR	GARCH	SV
UVaR	2836.899	1150.985	2525.38
UES	3505.298	1428.759	3140.247
LVaR	2554.215	1033.545	2262.439
LES	3183.424	1295.037	2840.514
IVaR	(2527.917, 2864.828)	(1022.619, 1162.588)	(2238.003, 2551.385)
IES	(3153.574, 3537.194)	(1282.636, 1442.01)	(2812.744, 3169.977)
BRNVaR	2693.042	1091.22	2391.514
BRNES	3341.207	1360.589	2987.386
VaR	768.9944	1049.765	1487.999
ES	931.4985	1316.447	1866.011

References

[1] Artzner P, Delbaen F, Eber JM, Heath D (1999) Coherent measures of risk. Mathematical Finance 9(3):203-228.

[2] Bhadra A, Datta J, Polson NG, Willard B (2019) Lasso meets horseshoe: a survey. Statistical Science 34(3):405-427.

[3] Carvalho CM, Polson NG, Scott JG (2009) Handling sparsity via the horseshoe. Journal of Machine Learning Research 5:73-80.

[4] Carvalho CM, Polson NG, Scott JG (2010) The horseshoe estimator for sparse signals. Biometrika 97:465-480.

[5] Chen Z, Epstein L (2002) Ambiguity, risk, and asset returns in continuous time. Econometrica 70(4):1403-1443.

[6] Cont R (2006) Model uncertainty and its impact on the pricing of derivative instruments. Mathematical Finance 16(3):519-547.

[7] Fan J., Yao Q (2003) Nonlinear time series: nonparametric and parametric methods. Springer, New York.

[8] Fouque J-P, Ren B (2014) Approximation for option prices under uncertain volatility. SIAM Journal on Financial Mathematics 5(1):360-383.

[9] Lau JW and Siu TK (2008) Modelling long-term investment returns via Bayesian infinite mixture time series models. Scandinavian Actuarial Journal 2008(4):243-282.

[10] Lo AY (1984) On a class of Bayesian nonparametric estimates: I. density estimates. Annals of Statistics 12(1):351-357.

[11] Peng S (2004) Nonlinear expectations, nonlinear evaluations and risk measures. In: Frittelli M, Runggaldier WJ (ed) Stochastic methods in finance. Lecture notes in mathematics 1856. Springer, New York, pp 165-138.

[12] Peng S (2006) G-expectation, G-Brownian motion and related stochastic calculus of Itô's type. In: Benth FE, Di Nunno G, Lindstrom T, Øksendal B, Zhang T (ed) The Abel Symposium 2005, Abel Symposia 2. Springer-Verlag, Berlin, pp 541-567.

[13] Polson NG, Scott JG (2010) Shrink globally, act locally: sparse Bayesian regularization and prediction. Bayesian Statistics 9:501-538.

[14] Polson NG, Scott JG (2012) Local shrinkage rules, Lévy processes and regularized regression. Journal of the Royal Statistical Society, Series B, 74:287-311.

[15] Polson NG, Scott JG, Windle J (2014) The Bayesian bridge. Journal of the Royal Statistical Society, Series B, 76(4):713-733.

[16] Polson NG, Sun L (2019) Bayesian l_0-regularized least squares. Applied Stochastic Models in Business and Industry 35(3):717-731.

[17] Rosazza Gianin E (2006) Risk measures via g-expectations. Insurance: Mathematics and Economics 39(1):19-34.

[18] Tong H (1990) Nonlinear time series: a dynamical system approach. Oxford University Press, Oxford.
